# Machinability of Basalt and Glass Fiber Hybrid Composites in Dry Drilling Using TiN/TiAlN-Coated Drill Bits

**DOI:** 10.3390/polym17162172

**Published:** 2025-08-08

**Authors:** Mehmet İskender Özsoy, Satılmış Ürgün, Sinan Fidan, Eser Yarar, Erman Güleç, Mustafa Özgür Bora

**Affiliations:** 1 Department of Mechanical Engineering, Faculty of Engineering, Sakarya University, Sakarya 54050, Türkiye; 2 Department of Aviation Electrics and Electronics, Faculty of Aeronautics and Astronautics, Kocaeli University, Kocaeli 41001, Türkiye; urgun@kocaeli.edu.tr; 3 Department of Airframe and Powerplant Maintenance, Faculty of Aeronautics and Astronautics, Kocaeli University, Kocaeli 41001, Türkiye; sfidan@kocaeli.edu.tr (S.F.); ozgur.bora@kocaeli.edu.tr (M.Ö.B.); 4 Department of Mechanical Engineering, Faculty of Engineering, Kocaeli University, Kocaeli 41380, Türkiye; eser.yarar@kocaeli.edu.tr; 5 Otokar Automotive Defense Industry Corp., Sakarya 54580, Türkiye; egulec@otokar.com.tr

**Keywords:** drilling, hybrid composites, drill-bit coatings, thrust force, hole quality, damage mechanisms

## Abstract

Drilling-induced damage in fiber-reinforced polymer composite materials was measured excavating four laminates, basalt (B_14_), glass (G_14_) and their two sandwich type hybrids (B_4_G_6_B_4_, G_4_B_6_G_4_), with 6 mm twist drills at 1520 revolutions per minute and 0.10 mm rev^−1^ under dry running with an uncoated high-speed steel (HSS-R), grind-coated high-speed steel (HSS-G) or physical vapor deposition-coated (high-speed steel coated with Titanium Nitride (TiN) and Titanium Aluminum Nitride (TiAlN)) drill bits. The hybrid sheets were deliberately incorporated to clarify how alternating basalt–glass architectures redistribute interlaminar stresses during drilling, while the hard, low-friction TiN and TiAlN ceramic coatings enhance cutting performance by forming a heat-resistant tribological barrier that lowers tool–workpiece adhesion, reduces interface temperature, and thereby suppresses thrust-induced delamination. Replacement of an uncoated, grind-coated, high-speed-steel drill (HSS-G) with the latter coats lowered the mechanical and thermal loads substantially: mean thrust fell from 79–94 N to 24–30 N, and peak workpiece temperatures from 112 °C to 74 °C. Accordingly, entry/exit oversize fell from 2.5–4.7% to under 0.6% and, from the surface, the SEM image displayed clean fiber severance rather than pull-out and matrix smear. By analysis of variance (ANOVA), 92.7% of the variance of thrust and 86.6% of that of temperature could be accounted for by the drill-bit factor, thus confirming that the coatings overwhelm the laminate structure and hybrid stacking simply redistribute, but cannot overcome, the former influence. Regression models and an artificial neural network optimized via meta-heuristic optimization foretold thrust, temperature and delamination with an R^2^ value of 0.94 or higher, providing an instant-screening device with which to explore industrial application. The work reveals TiAlN- and TiN-coated drills as financially competitive alternatives with which to achieve ±1% dimensional accuracy and minimum subsurface damage during multi-material composite machining.

## 1. Introduction

Fiber-reinforced polymer (FRP) composites have proven irrevocable in high-performance engineering structures because they possess exceptional specific strength, light weight, corrosion resistance, and controllable mechanical properties. Because of their benefits, they are highly demanded in critical sectors like aerospace, automotive, defense, and wind power [1,2,3,4,5,6,7]. Among FRP systems, hybrid composites comprising two or more fiber types embedded in a single polymer matrix are increasingly popular for their ability to synergistically exploit the merits of each fiber. In particular, basalt–glass hybrid composites have attracted significant attention due to their balanced combination of mechanical behavior, tribological performance, and cost-effectiveness [8,9,10,11,12,13]. Basalt fibers, which are extracted from natural volcanic stone, possess greater thermal stability, elastic modulus, and chemical resistance in acidic/alkaline mediums when contrasted with glass fiber, yet glass remains economical and exhibits excellent tensile strength [14,15]. By optimizing stacking sequences or weave structures, the inherent shortcomings related to delamination and thermal distress can be suppressed in hybrid composites. Basalt–glass fiber hybrid composites, in particular, are introduced as economically viable alternatives to traditional carbon fiber systems. Glass fibers are very popular because they are inexpensive, possess good tensile strength and chemical resistance, and yet remain economical and exhibit excellent tensile properties [15].

Rajendran et al. [16] studied the possibility of sugarcane biochar being used as the filler material for S-glass/polyester hybrid composite material and found the incorporation of the 10 wt% biochar loading to impart the composite with much elevated tensile strength (119 MPa), flexural strength (154 MPa), and impact resistance (45 MPa). The research pointed to the role of biochar to reduce void content and enhance stress distribution with the combination often optimized when manufactured with vacuum bagging procedures that exceeded traditional hand layup procedures through enhanced resin infiltration and minimization of air gaps. Recent work emphasizes the critical role of filler material to extend NFC properties. For example, industrial residue filler materials such as fly ash and red mud are exemplified to enhance mechanical strength when added to natural fiber-reinforced composite materials. Analogously, nanoparticle filler materials such as carbon nanotubes and graphene are able to upgrade thermal conductivity while sustaining the environmental friendliness of the composite materials. Chemical modifications tend to be important for the optimalization of fiber–matrix adhesion and composite performance as well as alkali and silane modifications [17]. The use of hybrid fiber architectures greatly enhances fatigue life to >2 million cycles at stress(es) +40% elevation relative to shell–core architecture as illustrated by Guo et al. [18], where the carbon/glass rod bars were uniformly dispersed and achieved >2 million cycles. Yu et al. [19] probed the submersion and alkaline rain exposure of carbon–glass hybrid fiber-reinforced polymer (HFRP) bars and found the thin coat of carbon fiber led to elevated water absorbency as well as diffusivity when exposed to weather relative to GFRP bars. Nonetheless, the thin film carbon fiber coat considerably elevated the interluminar shear strength retention when immersed in alkalic conditions by hindering alkalic ions’ reaction with internalized glass fiber.

Basalt fibers, extracted in situ from naturally deposited volcanic stone, possess greater thermal stability, elastic modulus, and chemical resistance in acidic and alkaline mediums when contrasted with glass fiber [15,20]. Because either fiber can enhance another’s properties when used together in either a stacked or interweaved medium, in this specific combination, they exhibit synergy and provide composites with greater impact behavior, interlaminar and shear strength, and dimensional stability and remain economically viable. Further, because using them together in this medium renders it possible for lamination sequences to be customized, it becomes feasible to texture the lamination so as to localize stiffness and enhance damage tolerance and diminish machinability-induced failure like delamination when they are drilled or machined. For this reason, basalt–glass composites are used increasingly in structural and fire-rated structural parts like interior airplanes skins, car underneath parts and blades in wind power systems where robustness and toughness in terms of structural behavior and manufacturing economy are most important.

Drilling is a significant post-processing operation for FRP composites, particularly in mechanically fastened applications. Despite this, the anisotropic and heterogeneous nature of the material predisposes it to machining-induced defects. Delamination significantly damages structural integrity and fatigue performance. Further damage mechanisms are fiber pull-out, due to incomplete fiber severance, and matrix smearing as a result of thermal softening and matrix adherence to the tool. Uncut fibers and hole ovality further compromise hole quality and dimensionally, resulting in fit-up and stress concentration issues. All this becomes worse when operating in dry drilling conditions, where temperatures close to the tool–workpiece interface can be in excess of the matrix glass transition temperature and thus lead to fiber–matrix adhesion weakening and interfacial debonding. Such defects compromise both mechanical performance and increase processing time and expense as a result of required secondary processing. Optimizing tool geometry, cutting and related parameters, and cooling methodologies becomes necessary in therefore improving drilling quality in hybrid and conventional FRP laminates [20,21,22]. There are numerous studies in which optimizing drilling parameters, tool materials and coatings were used in achieving minimal damage. In their work examining carbon/glass fiber hybrid composites, Patel et al. [15] examined the drilling in the presence of hybrid basalt–glass polyester composites using HSS drills as they vary in stacking sequence and found thrust force ranging between 72.6 and 109.3 N, and where the highest thrust force corresponded to where there were glass fiber appearances in the outer surfaces. They reported further that delamination resistance in symmetric G/B/G stacks was better compared to asymmetric stacks as a result of enhanced load distribution and fiber bridging in the region between the tool and laminate. In another related study, Yalçın et al. [20] examined depending and tool diameter and point angle and spindle speed effects in examining the aerospace-grade glass/epoxy composites as drilled in using newly developed in-house designed twist drill. They noted delamination increased in accordance with increased tool diameter and feed rate and where lower thrust force and delamination were obtained using 4 mm tool diameter and 900 rpm. Optimized configuration minimizing entry delamination factor from 1.30 down to 1.08 justified the effectiveness where in the use of customized geometry in minimizing damage. Drilling-induced thermal effects are also a major reason for subsurface damage, especially in dry conditions. CFRP/Ti stack drilling has been researched by Jia et al. [21], where it has been found that cutting temperature was as high as 280 °C and induced thermal softening of the polymer matrix and matrix–fiber debonding. It has been proven in the investigation that the exit delamination factor rose in proportion with temperature and thus highlighted the composite drilling necessity for thermal management.

Hybridization also helps in minimizing damage. Interlaminar shear strength and microcracking were reported by Chauhan and Bhushan [22] as enhanced in hybrid carbon fiber/epoxy composites with the reinforcement of nano-carbon black as compared to neat carbon laminates. They demonstrated through this investigation that the balancing in surface energy obtained during the process of hybridization helped in better drilling performance with 35% reduction in delamination. For fiber sequence and fiber orientation, multi-objective optimization during drilling in hybrid FRPs with different stacking sequences was performed by Dubey et al. [23]. Using feed rate (0.1–0.5 mm/rev) and cutting speed (60–150 m/min) as variables, they confirmed that least delamination and thrust force were experienced when outer layers were as basalt layers and inner as glass layers and this ascribed by the increased stiffness and resistance towards crack in the case when using basalt plies. Thrust force and torque in this case were reduced by 27% and 18%, respectively, as compared to the inverse layup.

Subagia et al. [24] studied conventional dry drilling of carbon/basalt hybrid composites using HSS tools and found delamination damage severe on the exit side due to burr formation and inadequate chip removal. It was found in the study that spindle speed greater than 1500 rpm and feed rate greater than 0.3 mm/rev significantly increased thrust force (up to 130 N) and promoted damage modes such as resin chipping, interlayer delamination, and thermal cracking. Durão et al. [25] studied damages provoked during drilling in glass and carbon fiber-polymer matrix hybrid composites with waste micro-inclusions such as silica particles, recycled carbon fiber dust, and cement. Experimental results verified that micro-particle inclusion enhanced the mechanical performance significantly, and flexural strength increased by approximately 23% in 40% volume fiber fraction composites and approximately 10% in 60% volume fiber fraction composites. More significantly, inclusion effects aided in minimizing drilling damage and improved hole quality, which indicates that inclusion of waste material boosts mechanical properties and promotes environmental sustainability in hybrid polymer matrix composites.

Yazman et al. [26] analyzed unidirectional CFRP, GFRP, and hybrid composite machinability in drilling, with attention to the effect of symmetrical stacking sequences in hybrids. Results were that carbon-dominated composites (C) certainly experienced the greatest thrust forces but the G/C and C/G hybrids had mid-values. Stacking carbon underneath had greater thrust forces but reduced exit delamination but the buildup increased the torque. Glass layers at the bottom increased torque. Peel-up delamination was highest in G/C hybrids and push-out delamination in neat GFRP composites. Ideal damage control was attained in C/G stacking balancing between machinability and integrity. High spindle speeds (4500 rpm) and low feed rates lowered delamination and surface texture in all setups. Kumar et al. [27] analyzed ramie-bamboo hybrid composite drilling performance reinforced using epoxy and nano-fillers (SiC and Al_2_O_3_). Results were that spindle speed had the most significant effect, and raising it (to 5500 rpm) reduced surface texture by 76.5% and delamination by 66.7%. Low feed rates (0.01 mm/rev) further reduced damage, and step drill bits were superior in achieving smooth surfaces and low delamination when compared with twist and core drills. Optimized characteristics—5500 rpm, 0.01 mm/rev feed, and step drill—obtained the best hole quality with profound implications for the significance of selecting parameters in natural fiber composite machining.

Raj et al. [28] studied thrust force generation in the process of drilling in natural fiber-reinforced hybrid composites (Abaca/Hemp and Hemp/Jute) using 90°, 100°, 105°, and 118° point angle twist drill bits. Results showed minimal thrust force (4.6 N) in Abaca/Hemp composites was seen at 3000 rpm, 0.05 mm/rev feed rate, and 90° point angle, and minimal thrust force (3.1 N) in Hemp/Jute composites was seen at 3000 rpm, 0.15 mm/rev, and 105° point angle. ANOVA reported a significant contribution of the drill point angle towards thrust force (64.93% in Abaca/Hemp, 75.39% in Hemp/Jute) followed by feed rate. Larger point angles increased friction and temperature but temperatures in the range below 45 °C confirmed hole quality. Pathak et al. [29] observed the behavior in drilling in carbon-Kevlar interyarn hybrid thermoplastic composites and monolithic carbon (CFPP) and Kevlar (KFPP) composites. Results confirmed the superiority in the performance of the brad spur drill (BSD) in comparison with conventional twist drill (CTD) and reduced the temperature in the process of drilling by 32.40%, thrust force by 29.73%, and delamination factor by 13.57% for the hybrid composite (CKFPP). Increased spindle speed increased temperature but decreased thrust force by matrix softening, and increased feed rates increased thrust force but decreased temperature. Delamination was minimal at moderate feed rates (30 mm/min). Microstructural analysis confirmed that BSD provided smoother surfaces with minimal number of defects. Performance in the hybrid composite was between CFPP and KFPP and balanced between stiffness and toughness. Optimum drilling conditions were confirmed through BSD at minimal feed rates and spindle speeds.

Rao et al. [30] studied the drilling behavior of carbon fabric/epoxy composites (CEC) filled with hexagonal boron nitride (h-BN) and molybdenum disulfide (MoS_2_) fillers. It was reported that filler-loaded composites reduced the temperature of drilling (maximum 24.7% in the case of MoS_2_ and 25.2% in the case of h-BN), surface roughness (minimum 0.78 µm in the case of MoS_2_), and delamination factor at exit (minimum DFexit of 1.0654 in the case of MoS_2_) when compared with neat CEC. Optimized parameters were in the form of high spindle speed (5500 rpm), low feed (0.03 mm/rev), and small diameter (4 mm) for the drill. SEM and C-scan studies confirmed increased surface integrity and minimal damage in fillers-loaded composites. Shahabaz et al. [31] studied the optimization of neat CFRP and hybrid CFRP nanocomposites (SiC and Al_2_O_3_) in the reduction in delamination and burrs during drilling. Results revealed that burr area and delamination in the case of nanocomposites were very low when compared with neat CFRP. Optimized drilling behavior was confirmed as consisting of high spindle speed (5500 rpm), low feed (0.01 mm/rev), and small diameter (4 mm) for the drill, in which the step drills were in the best category. Drill diameter and type were confirmed as the most significant factors using ANOVA.

Shanmugam et al. [32] examined the drilling characteristics of silane-coated sisal/redmud hybrid composites in thrust force, delamination, and surface roughness. It was determined that 118° point angle was best in minimizing thrust force and 90° angle in minimizing delamination. Spindle speed (1000 rpm) and feed rate (100 mm/min) were best in hole quality. Spindle speed (39.23%) and feed rate (27.51%) were significant factors in thrust force using ANOVA, and point angle (63.05%) controlled delamination. Spindle speed and feed rate were least significant in terms of affecting surface roughness, and optimum values were 135° point angle. Bukhari et al. [33] examined the behavior in drilling carbon–glass fiber composites using specially fabricated and designed six drills with unique geometries. After examining them, it was determined that Thrall 6 (135° and 90° point angle, 40° helix angle) was better in all properties and had least delamination and thrust force and enhanced hole quality (cylindricity, roundness). Optimum values were 640 rpm spindle speed and 0.08 mm/rev feed rate. Delamination was restrained at hole entry when it was detected.

Yarar et al. [34] examined machinability in glass (G_14_), basalt (B_14_), and hybrid (B_4_G_6_B_4_, G_4_B_6_G_4_) epoxy composites during drilling. Thrust force ranged between 65.8 N (B_14_ at 0.1 mm/rev) and 174.3 N (G_14_ at 0.3 mm/rev) with the hybrids in between. G14 experienced maximum temperatures (110–120 °C), and hybrids provided thermal loads below 90 °C. Feed rate was seen as the most significant factor (73.4%) in thrust force by analysis of variance (ANOVA), and material type (41.5%) regulated temperature. Symmetric G_4_B_6_G_4_ had dimensionally stable diameter ±2%, and Kar et al. [35] examined TiN, TiAlN, and TiCrN-coated tungsten carbide tool drilling characteristics in hybrid bio-polymer composites. Delamination was reduced 16.47% (front) and 13.95% (rear) and surface roughness enhanced 42.85% using TiN-coated tools, and TiCrN-coated tools restrained circularity error 28.10%. By using ANOVA, tool coating was seen as the most significant factor, followed by spindle speed and feed rate as significant factors. Optimum conditions for drilling (TiN-coated tool, 1800 rpm spindle speed, 0.12 mm/rev feed rate) significantly enhanced hole quality and validated the effectiveness of tool coatings in defect reduction and enhanced machinability in bio-composites. Haoua et al. [36] investigated drilling of hybrid stacks composed of CFRP, titanium, and aluminum alloys using an electric automated drilling unit (eADU). Due to the differing machinability of each layer, challenges such as delamination, fiber pullout, and burr formation arise. To address this, they developed a real-time material identification approach based on multi-sensor data fusion and a random forest machine learning model. By monitoring cutting forces, vibrations, lubrication parameters, and spindle/feed motor currents, the system accurately distinguishes material types during drilling. Biruk-Urban et al. [37] investigated drilling quality in thin glass fiber-reinforced polymer (GFRP) plates produced by vacuum bagging and an innovative vacuum mold pressing method. Their study focused on the effects of factors such as support opening width, fiber weight fraction, feed per tooth, and hole area on cutting force, delamination factor, and surface roughness. Using a statistical design of experiments and microscopy analysis, they found that feed per tooth and hole area significantly influenced delamination and surface roughness. Optimal hole quality in twill-based GFRP was achieved at a low feed rate of 0.04 mm/tooth and a support opening width of 55 mm. Sridhar et al. [38] conducted a systematic study on the effects of spindle speed and axial feed on drilling performance metrics including thrust force, machining temperature, surface roughness, hole size, burr size, and chip morphology. Their results indicated that higher spindle speeds (up to 4000 rev/min) combined with lower axial feeds (0.1 mm/rev) significantly reduced thrust force, while surface finish improved at higher feeds (0.4 mm/rev). Machining temperature increased with higher spindle speeds and lower feeds. Burr size was larger at hole exit than entry and increased with both spindle speed and feed. Moreover, higher axial feed and spindle speed improved chip breakability and evacuation.

Slamani et al. [39] assessed the influence of the material of drills in hole quality in jute/palm fiber-reinforced hybrid composites, accounting for delamination, circularity, and cylindricity. Variable feed rates and spindle speeds were used in experimenting with HSS, cobalt-coated HSS (HSS-Co5), and solid carbide drills. Experimental findings revealed that HSS drills were better and consistent in comparison with HSS-Co5 and solid carbide drills in delamination reduction and better geometrical accuracy with uncertainty reduction. Statistic analysis confirmed significant effects of feed rate and material of the drill in hole quality. Du et al. [40] analyzed the CF/PEEK composites’ drilling using the twist, brad, and dagger drills. Experimental findings revealed that the thrust force was reduced by 36.7% using the brad drill when it was utilized in comparison with the twist drill. However, the twist drill reduced the temperature by 23.9% when it was utilized in comparison with the dagger drill. The brad drill reduced burrs but introduced tearing damage, and the dagger drill introduced fewer delaminations but raised burrs since the temperatures were softening the PEEK matrix. It also obtained smooth hole walls using the brad drill, and in comparison, continued crimped chips were obtained using all the drills. Murgaiyan et al. [41] show that the addition of 1.5 wt.% of nanoclay to GLARE laminate enhances flexural strength (337 MPa) and interlaminar shear strength (16 MPa) through the enhancement of aluminum–fiber interfacial bonding. Conversely, high loading of nanoclay (2 wt.%) leads to particle agglomeration and reduces mechanical performance. The research also shows that the shape of the indenter is important on puncture resistance with flat indenters recording higher peak loads (11,369 kN) when compared to hemispherical indenters (6805 kN) for the 2 wt.% nanoclay-based composite.

Whereas previous work has extensively investigated monolithic composite drilling performance with composite types including carbon or glass fiber-reinforced polymers, there is a critical knowledge gap for the machinability of hybrid basalt–glass fiber-based composites. Research to date is largely predicated on particular fiber types or limited coating options (e.g., individually TiN or TiAlN), frequently to the detriment of the interaction of the synergistic action between the stacking sequence of the hybrids and the high-performance coatings on thrust force, thermal conductivity, and quality of the hole. There are not many studies that have employed extensive statistical and microscopic analyses to characterize coating performance with damage mechanisms for the architectures of hybrids. This study gives an extensive investigation into machinability in hybrid basalt–glass fiber-reinforced composites and the influence of drill-bit coatings (uncoated HSS, ground HSS, TiN, and TiAlN) in thrust force, drilling temperature, and hole quality. Unlike previous studies that primarily concerned themselves with monolithic composites or limited types of coatings, this study systematically evaluates four distinct composite configurations (B_14_, G_14_, B_4_G_6_B_4_, G_4_B_6_G_4_) with standardized drilling conditions and provides comparative thermal and mechanical behavior analysis. The engineered B_4_G_6_B_4_ and G_4_B_6_G_4_ laminate selections were specially picked to optimally test tool–composite interaction. The two are symmetric sandwich-type structures and by stacking the outer and inner layers according to fiber type, they allow for the distinction of significant parameters such as delamination, temperature distribution, and tool wear that are potentially encountered while drilling. These configurations allow for the clear observation of the role of the fiber type in the outer layer during the initial tool contact, while the effects of the fibers in the inner layer on thermal dissipation and matrix stability were evaluated. The effects of similar hybrid layouts (e.g., basalt on the outside/glass on the inside, or vice versa) on drilling performance have been previously demonstrated in the literature [23,34]. The use of these structures contributes to balancing mechanical load distribution, reducing tool forces, and achieving a more controlled metal removal process. It is also known that symmetric structures limit internal stress accumulation, thus increasing statistical consistency. Therefore, the chosen hybrid structure arrangements reflect an approach that is justified from both scientific and practical perspectives and is consistent with the literature.

Advanced characterization techniques, i.e., SEM and infrared thermography, discern the role of coating-created friction reduction in matrix integrity retention, particularly in glass-rich layers. Furthermore, factorial analysis determines the thrust force effect, which supersedes material composition. By using the combination of empirical information and statistical and microscopic analysis, this study presents new findings towards optimizing tool-choice in hybrid composites and fills the critical lacuna in the literature where previous work primarily concerned itself with carbon or glass fiber in individual roles. Industry guidelines as the outcome of the study present workable recommendations towards precision and longevity improvement in drilling through sustainable, performant hybrid composites. Unlike previous work, this research systematically assesses four different composite configurations—monolithic basalt (B_14_), monolithic glass (G_14_), and two hybrid stacks (B_4_G_6_B_4_, G_4_B_6_G_4_)—under similar drilling conditions to differentiate the tool-coating effects. State-of-the-art microscopy tools like scanning electron microscopy (SEM), infrared thermography are utilized to differentiate coating-driven friction reduction and matrix integrity preservation, especially for the glass-rich layers. Factorial analysis (ANOVA), too, quantitatively proves that the drill-bit coatings are responsible for more than 90% of the variance in the thrust force and temperature as against laminate structure-based variations, thereby relegating the latter to the sidelines. This research not only fills the gap in the machinability of the hybrid composite but also presents industry-ready guidelines on how to achieve precision drilling with TiN/TiAlN-coated tools. Moreover, by systematically contrasting TiN- and TiAlN-coated HSS drills with their uncoated counterparts under identical feed–speed conditions across symmetric basalt–glass hybrids, the present work is the first to quantify coating-by-stacking-sequence synergies, thereby extending cutting-tool coating performance maps, previously validated only for monolithic CFRP or GFRP, to multi-material laminate architectures. Practical implications of the research work—like the achievement of ±1% dimensional tolerance and the reduction in subsurface damage with the help of TiAlN/TiN-coated drills—represent tangible progress beyond the prevailing solutions that commonly fail to counter delamination and thermal damage in the hybrid composite.

## 2. Materials and Methods

### 2.1. Materials

**Figure 1:**
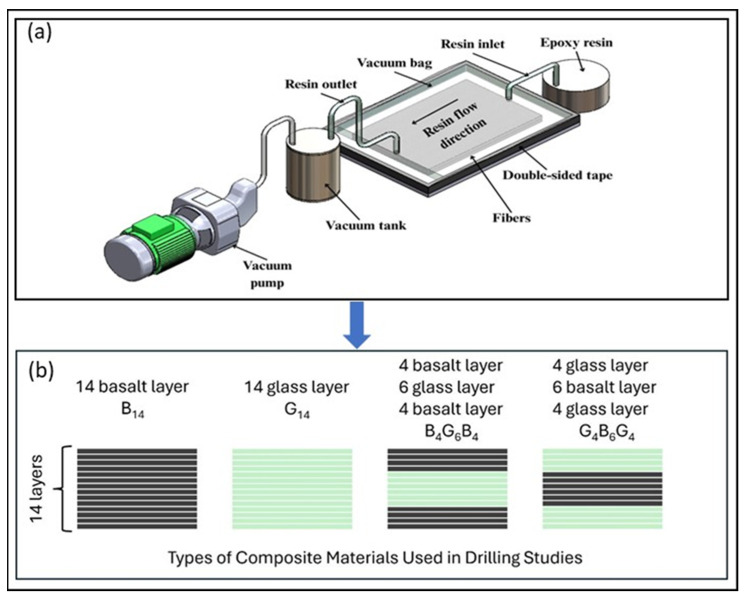
Schematic representation of manufacturing method (**a**) and composite laminates used in drilling experiments (**b**).

In this study, the machinability of four different fiber-reinforced polymer composite laminates was investigated. This study included two monolithic laminates fabricated exclusively from either basalt or glass woven fabrics, as well as two multi-layered sandwich type hybrid configurations that integrated both types of reinforcement in alternating sequences such as B_14_, a 14-layer basalt fiber-reinforced composite, G_14_, a 14-layer glass fiber-reinforced composite, and basalt and glass fiber hybrids such as B_4_G_6_B_4_ and G_4_B_6_G_4_. Plain-weave glass and basalt fiber cloths, having an areal weight of 200 g/m^2^, acted as the reinforcement materials and were supplied by Dost Kimya Company (Istanbul, Türkiye). The polymer matrix system used was the Biresin Sika CR80 epoxy resin with the CH 80-2 hardener (Sika Österreich GmbH, Bludenz, Austria), which formed a thermosetting network during curing. According to the supplier’s information, the material properties are as follows: Epoxy has a density of 1.13 g/cm^3^, a tensile strength of 83 MPa, and an elastic modulus of 2.9 GPa. Glass fiber has a tensile strength of 2306 MPa, an elastic modulus of 81.5 GPa, and a density of 2.59 g/cm^3^, while basalt fiber has a tensile strength of 3170 MPa, an elastic modulus of 89 GPa, and a density of 2.75 g/cm^3^. Figure 1 shows the manufacturing methods and produced composite laminates. Vacuum-assisted resin transfer molding was used to manufacture the laminates with a fiber/reinforcement ratio of 45%. Processing included the casting of the laminates under controlled environmental conditions and post-curing (60 °C, 4 h) in order to offer higher density of cross-links. Hybrid lay-ups changed with the positioning of the type of the fibers in the stacking sequence, allowing an assessment of how outer and inner configurations of the fiber at the stacking sequence level influence the fabric response during machining. Specimens were precisely delineated with water jet cutting to obtain dimensional consistency with the aim of enabling further characterization.

### 2.2. Experimental Setup

**Figure 2:**
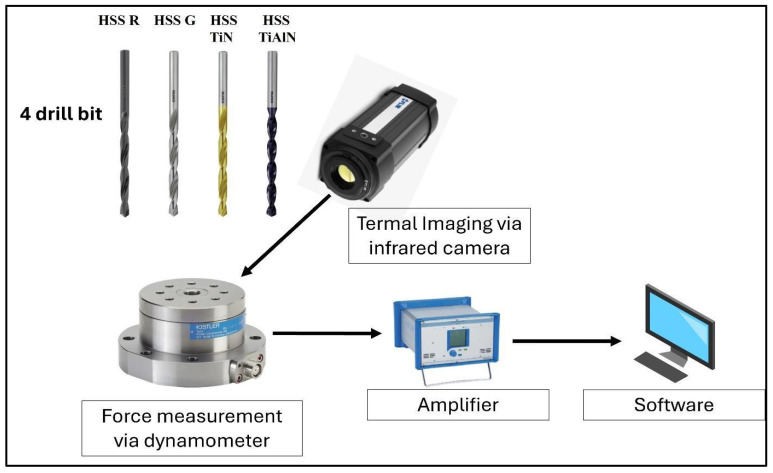
Experimental setup used in drilling tests.

All tests were performed in a dry environment utilizing a Toss United TU5032B vertical drilling machine, which delivered repeatable results with steady axial feed and spindle stability [42]. For assessing the influence of tool properties on drilling effectiveness, four specific types of 6 mm-diameter twist drills were used: uncoated rolled high-speed steel (HSS-R), uncoated ground high-speed steel (HSS-G), TiN-coated HSS, and TiAlN-coated HSS as seen in Figure 2. They are widely recognized in the literature as capable of reducing friction, enhancing temperature resistance, and extending tool longevity when machining composite materials whose abrasive fibers and heterogeneous structure accelerate tool wear and thermal failure.

### 2.3. Testing Procedures and Measurements

All trials had pre-set cutting conditions at 1520 rpm spindle speed and 0.1 mm rev^−1^ feed rate. Thrust force, employed to evaluate tool–workpiece interaction and delamination potential, was measured with a Kistler 9272 (Kistler, Winterthur, Switzerland) piezoelectric dynamometer connected to a Kistler 5070A signal-conditioning unit, while data logging was handled by Dyno Ware software (version 2825D-02), which provided high-resolution force traces throughout each drilling cycle. For thermal analysis, a FLIR A325sc (FLIR Systems, Inc., Wilsonville, OR, USA) infrared camera was positioned 50 cm above the specimen at a 60° angle and captured images immediately after tool exit for a standardized period, and temperature fields around the drill and hole perimeter were processed with FLIR Research IR software (version 1.2.10173.1002). The thermal analysis employed an average emissivity value of 0.95, which was carefully determined through extensive calibration tests involving heated drill tools and composite specimens. Post-drilling examinations included delamination and hole-quality measurements via a surface profilometer, together with subsurface damage inspection at the hole wall using scanning electron microscopy. The damage topography maps were obtained from full-field three-dimensional height data acquired with a Nanovea PS50 laser profilometer (Nanovea, Irvine, CA, USA); the resulting point clouds were processed in Mountains Technology DigitalSurf Software (version 6.2.7487), and rendered as true-color height maps with a ±60 µm z-scale to visualise entrance and exit side material loss. These integrated measurements provide a comprehensive framework for understanding the interplay among drill-bit type, coating, and laminate architecture during high-performance machining.

## 3. Results

Table 1 compiles the experimental findings from machining four different composite laminates, B_14_, G_14_, B_4_G_6_B_4_, and G_4_B_6_G_4_, under four different tool types: HSS-R, HSS-G, HSS-TiN, and HSS-TiAlN (RUKO GmbH, Holzgerlingen, Germany). For the material–tool couple, average thrust force, maximum drilling temperature, drill temperature, and surface temperature of the material are listed. Overall observation of the data shows that HSS-G registered the maximum thrust force and thermal output in all materials studied. Specifically, the maximum thrust force (94.17 N) and among the maximum temperatures (111.9 °C) registered its operation in cutting the glass fiber-dominant structure, G_14_, under HSS-G. This evidence shows extreme frictional heating and tool–material examples, notably in glass fiber-predominant laminates. Likewise, B_4_G_6_B_4_ and B_14_ registered the thrust forces greater than 79 N and surface temperatures higher than 100 °C, respectively, during their cutting with the latter tool. Conversely, the work with the minimum thrust force and temperature was registered on processing with HSS-TiN and HSS-TiAlN-based cutting tools, respectively. For instance, B_14_ processed with the former gave the least thrust force (24.19 N), in addition to the maximum surface temperature during drilling (70.3 °C). The former was followed closely, evidencing comparable behavior. The cutting tools with the coat, apart from minimizing the cutting force, restricted the grainage of the accumulating heat both in the drill and workpiece, respectively. Another observed pattern is that the former laminate, namely, the G_14_ consistently demanded the higher thrust force compared to the rest, especially under HSS-R and HSS-G, which is an indication of the existing translucency of glass fiber and its consequences on the loading of the cutting tools. Conversely, the laminate, whose structure is hybrid, namely, the G_4_B_6_G_4_, registered comparatively minimal force under the application of the coat tools, an indication of the balanced stacking sequence’s influence on the machinability of the structure, respectively.

### 3.1. Thrust Forces

**Figure 3:**
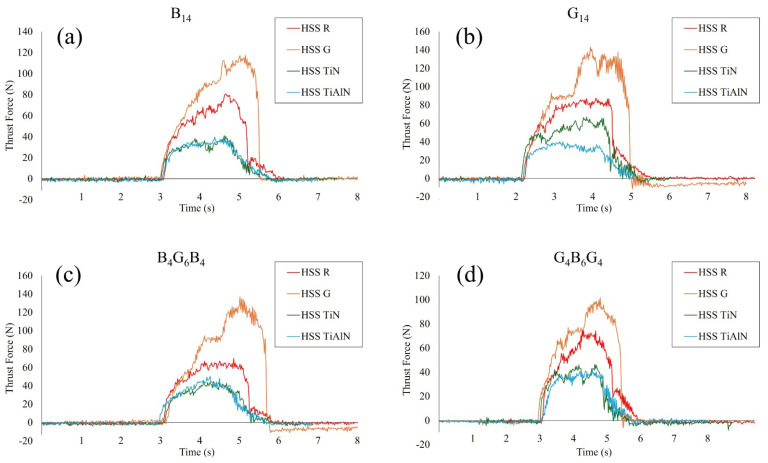
Thrust force graphs plotted for different drill bits for all composites.

The thrust force graphs, Figure 3, obtained from drilling G_4_B_6_G_4_, B_14_, B_4_G_6_B_4_, and G_14_ composite laminates using HSS-G, HSS-R, TiN-coated, and TiAlN-coated tools reveal systematic relationships between tool type, coating, and laminate composition. HSS-G generated the highest thrust forces in all laminates, followed by HSS-R. This correlates with the absence of wear-resistant coating and consequent rise in friction and thermal softening during cutting—a phenomenon extensively reported, especially in uncoated HSS tools drilling FRP composites [43,44,45]. TiN- and TiAlN-coated tools consistently registered substantially lower thrust values in most laminates, which is consistent with results demonstrating that coatings enhance surface hardness and thermal resistance, thereby effectively decreasing cutting resistance in basalt and glass fiber composites. Thrust force curves of the G_4_B_6_G_4_B, B_14_, and B_4_G_6_B_4_ laminates registered sharp increases during drill entry followed by flat spots during the principal drill phase with TiN and TiAlN tools. Writings refer to corresponding behavior, which shows stable cutting action and efficient chip removal in hybrid and basalt fiber composites with the use of coated tools. The TiAlN, consisting of purely glass fiber matrix, G_14_ registered lower thrust force values compared to TiN, which is an indicator of its enhanced bearing and thermal stability—features demonstrated to counteract the higher abrasivity of purely glass fiber environments [46]. The HSS G drill demonstrated an uninterrupted and continuously growing increase in drilling thrust force irrespective of the type of laminate. This indicates unstable cutting behavior and sustained cutting-tool wear, likely augmented due to inefficient collages and ineffective chip removal. Unlike the coated ones, the HSS-G is unable to provide an uninterrupted cutting edge, hence higher mechanical resistance with the advance of drillings. Considering the laminate-specific findings, the lowest average thrust force values registered belonged to the G_4_B_6_G_4_ laminate with all the different types of tools used. This is attributed to its balanced hybrid structure, which allows equitable loading and smoother engagement of the tool during drillings. The B_14_ and B_4_G_6_B_4_ laminates followed with moderate force reading due to basalt fiber loading, which elicits higher cutting resistance, albeit in an inferior configuration to that of the G_14_ laminate. The latter laminate registered the maximum average thrust force values of all the laminates used. This behavior is attributed to the abrasive quality of the glass fiber and the fact that it lacks the protective layering structure that helps to distribute drill stresses, hence it is the most challenging with regard to machinability. The significant fluctuation of thrust force with the different laminate configurations is not just because of the drill configuration and the coating but is also because of inherent material removal mechanisms as stipulated by the laminate configuration. For the hybrid stacks B_4_G_6_B_4_ and G_4_B_6_G_4_, the alternate stiff basalt and flexible glass fiber plies cause gradual transitions of the cutting resistances. The nature of the outermost type of fiber dominantly influences the tool–material engagement at the inception: the basalt layers exhibit higher resistance with their higher modulus while the glass layers exhibit more easily deformable nature to cause the chip shape and energy dissipation. Additionally, interfacial toughness between fiber–matrix plays the same role. The reduced interlaminar fracture toughness, particularly the glass-rich configurations, causes interfacial debonding along with fiber pull-out to enhance the thrust force. In return, the high interfacial strength for the basalt-rich configurations enables the clean shearing with less requirements for thrust. Additionally, the fiber orientation—all the specimens based on woven fabric-based configuration—causes oscillatory fiber engagement angles while drilling. Such oscillations influence the chip shape as well as the local cutting stress with particular reference to the uncoated tools where friction heat further destroys the matrix integrity. As such the trend of the thrust force observed for the hybrid configurations is the sum effect of fiber–matrix interaction, interfacial adhesion quality and the fiber sequence that governs the interlocal energy dissipation mechanisms as well as the stiffness as the drilling takes place.

### 3.2. Delamination and Hole Quality

**Figure 4:**
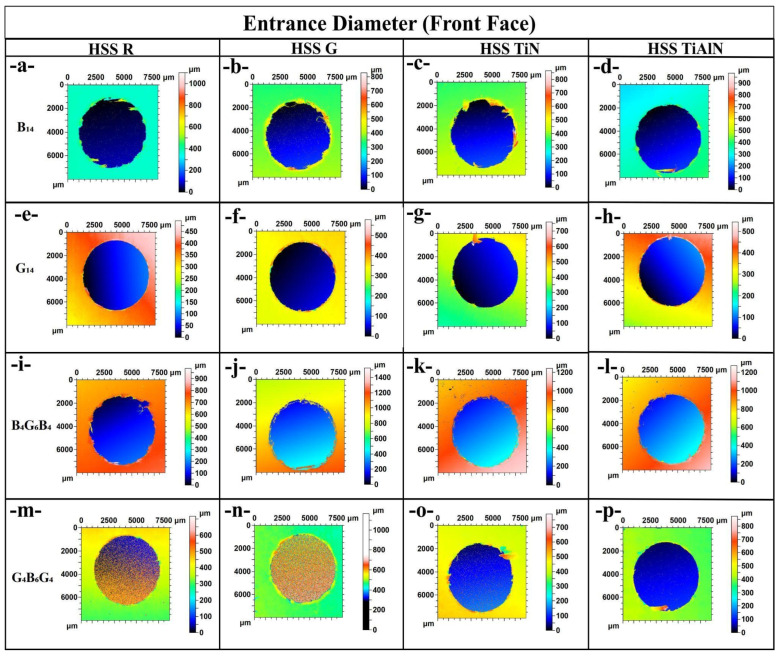
Entrance diameter comparison of holes with various drill bits: (**a**) B_14_: HSS-R, (**b**) B_14_: HSS-G, (**c**) B_14_: HSS-TiN, (**d**) B_14_: HSS TiAlN, (**e**) G_14_: HSS R, (**f**) G_14_: HSS-G, (**g**) G_14_: HSS TiN, (**h**) G_14_: HSS- TiAlN, (**i**) B_4_G_6_B_4_: HSS-R, (**j**) B_4_G_6_B_4_: HSS-G, (**k**) B_4_G_6_B_4_: HSS-TiN, (**l**) B_4_G_6_B_4_: HSS-TiAlN, (**m**) G_4_B_6_G_4_: HSS-R, (**n**) G_4_B_6_G_4_: HSS G, (**o**) G_4_B_6_G_4_: HSS-TiN, and (**p**) G_4_B_6_G_4_: HSS-TiAlN.

Figure 4 provides a vivid topographic snapshot of entrance-side damage and dimensional accuracy for every material–tool combination, and the sequence of images through plates traces how both laminate architecture and drill-bit design conspire to shape hole quality. In the basalt-rich B_14_ panel, Figure 4a (HSS-R) already shows a ragged, under-filled periphery, but the darker blue core confirms an entrance diameter of approximately 6050 µm, only 0.8% oversize; switching to the ground drill in Figure 4b exaggerates the scalloped lip and pushes the span to approximately 6150 µm, echoing the 79.9 N thrust and 111 °C peak temperature that this bit generated in Table 1 and linking the fiery palette around the rim to thermally driven matrix softening. Coated tools reverse the trend; Figure 4c (TiN) and Figure 4d (TiAlN) present nearly perfect circles with crisp edges and a chilled green–cyan backdrop, matching their sub-5950 µm diameters and low 70–69 °C drilling temperatures, evidence that PVD films suppress frictional heating and fiber breakout. The all-glass G_14_ laminate in images Figure 4e–h magnifies every cause–effect pair: the compliant fibers allow HSS G in Figure 4f to balloon the entrance to 6200 µm, over 3% oversize, leaving a pronounced orange halo of debris, whereas TiAlN in Figure 4h reins the hole back to 5970 µm and restores a clean blue interior. Hybrid lay-ups temper both extremes; in B_4_G_6_B_4_, images Figure 4i,j reveal that alternating plies blunt the jagged cavities seen in pure basalt, limiting oversize to 6080 µm for HSS R and 6180 µm for HSS G, while coated counterparts in Figure 4k,l again fall near 5980 µm and 5960 µm with markedly smoother borders. The reciprocal hybrid G_4_B_6_G_4_ follows suit, Figure 4m through Figure 4p, but tells the same cautionary tale; HSS-G in Figure 4n lifts thrust to nearly 70 N and paints a rough yellow fringe at 6170 µm, whereas TiN and TiAlN in Figure 4o,p produce tight, blue-green circles just under 5960 µm with virtually no fiber pull-out. Taken together, sub-figures Figure 4a–p converts raw numbers into color-coded anatomy lessons; the ground HSS G bit consistently marries the highest thrust and temperature to the widest, most damaged entrances, uncoated HSS-R fares slightly better but still displays micro-chipping, and both TiN and TiAlN coatings repeatedly shrink diameters by 170–230 µm while erasing peripheral delamination, effects that become more dramatic as laminate stiffness drops from basalt to glass. These correlations confirm that entrance-edge integrity is dictated first by the drill-bit frictional regime and second by the fiber–matrix compliance of the host laminate, with coated tools providing an indispensable thermal and mechanical buffer across all architectures.

**Figure 5:**
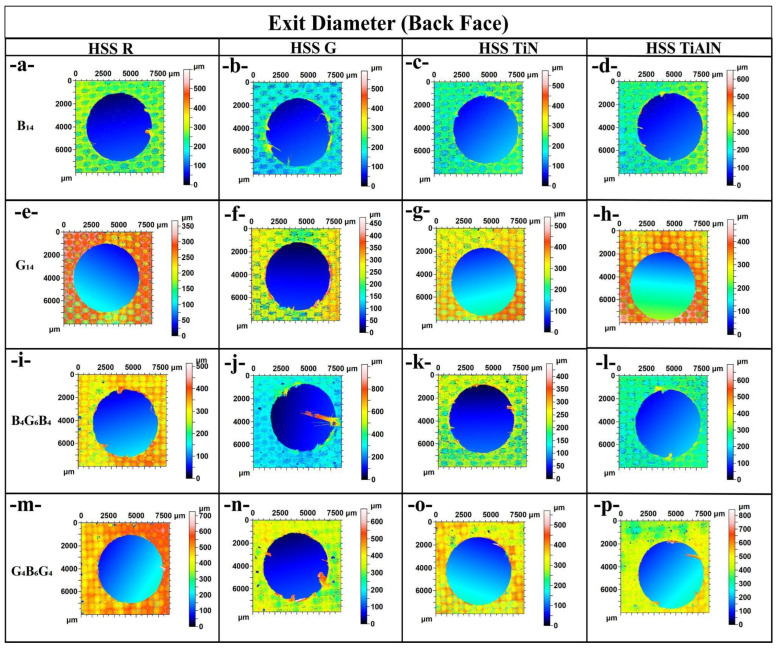
Exit diameter comparison of holes with various drill bits: (**a**) B_14_: HSS-R, (**b**) B_14_: HSS-G, (**c**) B_14_: HSS-TiN, (**d**) B_14_: HSS-TiAlN, (**e**) G_14_: HSS-R, (**f**) G_14_: HSS-G, (**g**) G_14_: HSS-TiN, (**h**) G_14_: HSS-TiAlN, (**i**) B_4_G_6_B_4_: HSS-R, (**j**) B_4_G_6_B_4_:-HSS G, (**k**) B_4_G_6_B_4_:-HSS TiN, (**l**) B_4_G_6_B_4_: HSS-TiAlN, (**m**) G_4_B_6_G_4_: HSS-R, (**n**) G_4_B_6_G_4_: HSS-G, (**o**) G_4_B_6_G_4_: HSS-TiN, and (**p**) G_4_B_6_G_4_: HSS-TiAlN.

Figure 5 translates the numeric exit-diameter data of Table 1 into color-coded morphology, letting us watch damage accumulate as the drill emerges from each laminate. In Figure 5a the uncoated HSS-R leaves the basalt-dominant B_14_ with a smooth blue interior bordered by a thin green–yellow ring, the 6120 µm span equating to a modest +2.0% oversize. Swapping to the ground bit in Figure 5b injects 79.9 N of thrust, tears a jagged orange plume and stretches the hole to 6230 µm, or +3.8%. Coated tools reverse the narrative; Figure 5c (TiN) and Figure 5d (TiAlN) cool the palette to cyan and restrain diameters to 6030 µm and 6020 µm, barely +0.5% and +0.3% above nominal. The all-glass G_14_ stack amplifies every contrast, Figure 5e shows HSS R climbing to 6180 µm (+3.0%) amid a red chequerboard of matrix smearing, while Figure 5–f lets the aggressive HSS G surge to 6280 µm (+4.7%) and carve ragged exit lips; yet Figure 5g,h prove that TiN and TiAlN can still corral the spread to 6040–6050 µm, the cooler greens signaling lower interlaminar shear rupture. Hybrid B_4_G_6_B_4_ moderates both extremes. In Figure 5i HSS-R opens to 6140 µm (+2.3%) with a peppering of orange pits, Figure 5j shows HSS G at 6250 µm (+4.2%) accompanied by a spray of breakout shards, whereas Figure 5k,l return to calm cyan rims at 6040 µm and 6030 µm. The reciprocal hybrid G_4_B_6_G_4_ in Figure 5m through Figure 5p repeats the pattern. HSS R exits at 6130 µm (+2.2%) with mild fiber pull-out, HSS G in Figure 5n drives thrust to 69.8 N and blows out a yellow-red crescent at 6240 µm (+4.0%), and the coated drills in Figure 5o (6020 µm) and Figure 5p (6010 µm) seal the back face almost flush with nominal size, leaving only faint green tracers of matrix recession. Across subfigures Figure 5a–p the message is unequivocal: exit-edge integrity tracks the frictional–thermal regime of the drill, with ground HSS-G consistently pairing the highest thrust and temperature with the largest, most delaminated openings, uncoated HSS-R offering modest improvement, and TiN/TiAlN coatings suppressing oversize by approximately 170–270 µm while erasing feathering and fiber peel-back, effects that intensify as laminate compliance rises from basalt to glass and are tempered but not eliminated by hybrid lay-ups. Comparisons were carried out between TiN- and TiAlN-coated and uncoated drills in the same dry drilling conditions [14]. TiN coats were seen to significantly reduce tool wear, drilling temperature, and delamination at the exit side, and the highest delamination factors were reduced from 1.52 (uncoated) to 1.21 (TiN-coated). They emphasized the importance of wear resistance and coating integrity in suppressing surface damage and improving hole dimensional accuracy. As stated in ref. [47], HSS (high-speed steel) and carbide cutting tools without coatings, with the same geometry and two types of cutting conditions (n = 1500 rpm, fn = 0.05 and 0.1 mm/rev) were used. A Sololite-type wooden backing plate was used to aid in reducing delamination. The results show that the additional support plates significantly reduced delamination by up to 80% both at the material inlet and especially at the drill hole outlet. The use of a lower feed rate (fn = 0.05 mm/rev) per tooth was shown to have a significant effect on reducing the delamination of bio composite materials with flax fibers, which are generally known to be difficult to machine. The carbide cutting tool shows significantly better results both in terms of its wear and in terms of delamination of the bio composite material. Saw drills and core drills produce less delamination than twist drills by distributing the drilling thrust towards the hole periphery. Delamination can be effectively reduced or eliminated by slowing down the feed rate when approaching the exit and by using back-up plates to support and counteract the deflection of the composite laminate leading to exit side delamination [48]. Slamani et al. [49] stated that the results reveal significant differences in delamination factors between the entrance (jute fibers) and exit (palm fibers) under varying machining conditions. Quantitative analysis reveals that optimal conditions for minimizing delamination occur at a spindle speed of 2388 rpm and feed rate of 0.04 mm/rev, achieving delamination factors of 1.121 (entrance) and 1.069 (exit). These findings emphasize the critical role of machining parameters in controlling drilling defects and improving the integrity of hybrid composite materials.

**Figure 6:**
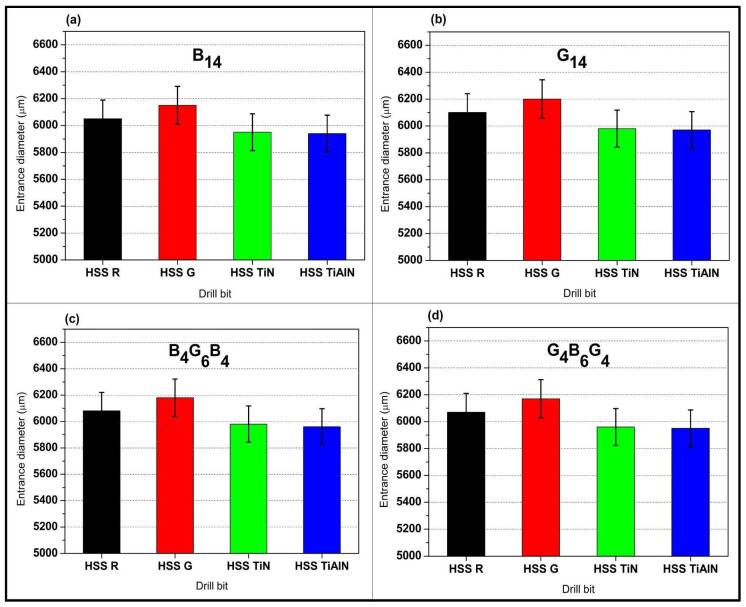
Entrance diameter comparison graphs plotted for different drill bits: (**a**) B_14_, (**b**) G_14_, (**c**) B_4_G_6_B_4_, and (**d**) G_4_B_6_G_4_ (error ± 2.3%).

Figure 6a focuses on the basalt-dominant B_14_ laminate and shows that the ground HSS G drill inflates the entrance to approximately 6150 µm, nearly 2.5% above the nominal 6 mm, whereas the roll-forged HSS R hovers near 6050 µm and both coated variants (TiN at 5950 µm and TiAlN at 5940 µm) actually undershoot the target, illustrating how low-friction coatings temper thermal expansion at entry. Figure 6b shifts to the all-glass G_14_ stack, and the same ordering persists but with an even wider spread. HSS G peaks at 6200 µm while TiAlN reins in the hole to 5970 µm, highlighting the greater compliance of glass fibers that magnifies tool-induced dilation. In the hybrid B_4_G_6_B_4_ panel of Figure 6c, entrance diameters fall between the extremes of the monolithic laminates, 6180 µm for HSS G and 5960 µm for TiAlN, suggesting that the alternating ply sequence damps the thermal–mechanical load transferred from the cutting edge. Figure 6d, depicting the reciprocal hybrid G_4_B_6_G_4_, confirms this moderation effect; although HSS G again leads at 6170 µm, both coated bits restrain oversize to approximately 5960 µm, underscoring that PVD coatings consistently deliver sub-2% dimensional error irrespective of lay-up. Collectively, the four subfigures demonstrate that entrance accuracy is ruled first by drill-bit condition—ground edges promote chip flow but sacrifice tolerance, while laminate architecture modulates, yet never eclipses, the benefit conferred by TiN and TiAlN coatings, as quantified in Table 1.

The comparisons of the entry diameters from Figure 6 demonstrate obvious advantages of the hybrids (B_4_G_6_B_4_ and G_4_B_6_G_4_) over monolithic systems (B_14_ and G_14_). As the stacks of singles with maximal responses, i.e., G_14_’s exaggerated oversize (6200 µm with HSS-G) through the abrasiveness of the glass fiber and B_14_’s thermally driven enlarging (6150 µm), the hybrids exhibit intermediate but more level responses. For instance, B_4_G_6_B_4_’s basalt-dominated exterior layers act to lessen initial tool engagement stresses through reduced entry oversize to 6180 µm (HSS-G), while G_4_B_6_G_4_’s exterior layers rich with glass allow smoother chip evacuation with restricted oversize to 6170 µm. It is the levelling action that is the product of the oscillatory ply flexibility of the hybrid: the basalt plies resist deformation and the glass plies absorb energy with the overall effect of diminishing the maximum stress on tool entry. It is to be noted that the tool coatings (TiN/TiAlN) augment this synergism additionally with the hybrids to reach nearly nominal diameters (5960–5970).

**Figure 7:**
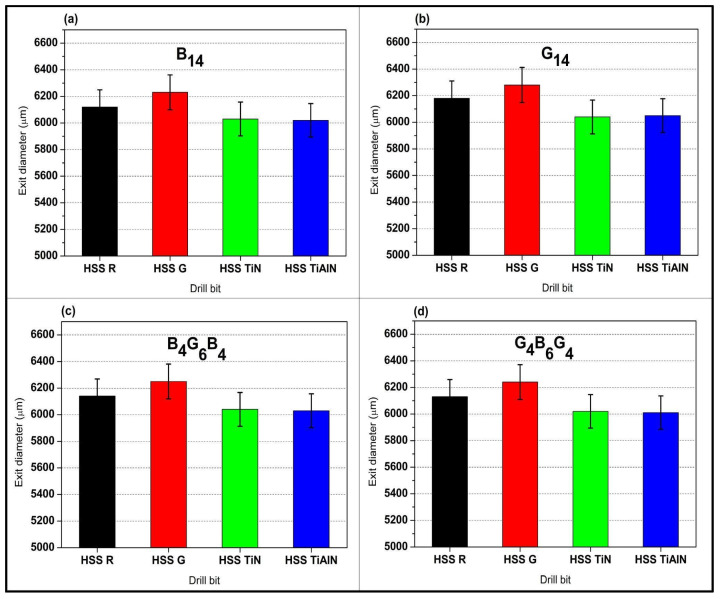
Exit diameter comparison graphs plotted for different drill bits: (**a**) B_14_, (**b**) G_14_, (**c**) B_4_G_6_B_4_, and (**d**) G_4_B_6_G_4_ (error ± 2.1%).

Figure 7a tracks the same B_14_ laminate at exit and reveals how accumulated thrust and heat exacerbate oversize; HSS-G enlarges the hole to 6230 µm (3.8%), whereas TiAlN limits it to 6020 µm, just 0.3% above nominal. Figure 7b shows this divergence widen in G_14_, where the more pliant glass allows HSS G to reach 6280 µm, yet the coated drills still corral the diameter around 6040–6050 µm. The hybrid B_4_G_6_B_4_ in Figure 7c again exhibits intermediate behavior, HSS-G at 6250 µm versus TiAlN at 6030 µm, reflecting the crack-arrest capability of alternating fibers that restrains delamination. Finally, Figure 7d confirms the trend for G_4_B_6_G_4_; although HSS-G exits at 6240 µm, both TiN and TiAlN keep expansion to approximately 6020–6010 µm, well within the ±2.1% experimental uncertainty reported in Figure 7. These four exit-side views underscore that coating-induced reductions in friction and thermal load are paramount for dimensional fidelity, especially in glass-rich assemblies where the synergy of higher thrust forces and lower interlaminar strength would otherwise exaggerate delamination and elastic spring-back.

Exit diameter tendencies in Figure 7 highlight the better damage tolerance of hybrids relative to monolithic stacks. Single-fiber systems demonstrate amplified exit-side delamination with G_14_ up to 6280 µm (HSS-G) through the low interlaminar strength of glass fibers and B_14_ at 6230 µm as the brittle failure of the basalt. Conversely, hybrids B_4_G_6_B_4_ and G_4_B_6_G_4_ demonstrate smaller oversize (6240–6250 µm with HSS-G), for which we attribute the arrest of cracks across basalt–glass interfaces and load partitioning. The symmetrical stacking sequence of hybrids such as the basalt–glass–basalt sequence for the B_4_G_6_B_4_ configuration keeps delamination to the inside plies, where it cannot catastrophically breakout. Coated tools take advantage of the architecture: the TiAlN prohibits exit oversize to ≤6030 µm in the hybrids as the thermal barrier role of the coating suppresses matrix softening at essential exit-phase loads. This combination between the hybrid architecture and high-performance coatings does not occur with the monolithic fiber laminas where thermal and load-mechanics focus into homogeneous ply interfaces.

### 3.3. Hole-Wall SEM Damage Analysis

**Figure 8:**
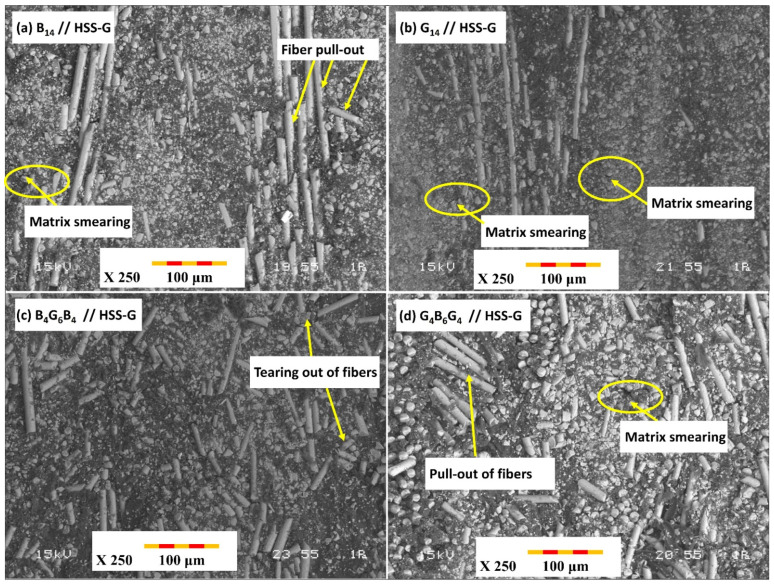
SEM images of the drilled hole cross-sections of the composites using the ground HSS-G drill bit (**a**) B_14_, (**b**) G_14_, (**c**) B_4_G_6_B_4_, (**d**) G_4_B_6_G_4_.

The SEM analyses on cross-sections of hole cutting by using TiAlN-coated HSS drill bit reveal substantially better-quality surfaces and minimum damage features on all three GFRP materials (Figure 8). SEM micrographs of samples performed after drilling tests using HSS (HSS-G) drill bit reveal essential information on magnitude of thermal and mechanical damage inflicted in each GFRP material (Figure 8). SEM photograph of B_14_ GFRP (Figure 8a) cut by HSS (HSS G) drill reveals excessive break-up of fibers and matrix cracking around hole periphery. This matches the highest captured thrust force (79.9 N) and corresponding max temperature (111.1 °C) for this tool–material couple. High thermal loading is suspected to have softened the matrix and resulted in debonding on interface, thus subsequent pull-out of fibers and delamination between plies. Profilometer results also indicated oversize hole cutting diameters on entry (6150 µm) and on exit (6230 µm), and this validates excessive delamination and thermal and mechanical damages. In G_14_ GFRP (Figure 8b), HSS-G drill resulted in excessive matrix smear and widespread break-out of fibers. SEM displays rough edges and thermal deterioration artifacts, and these are in agreement with highest force (94.17 N) and highest temperature (111.9 °C) captured among samples. Greater glass fibers’ compliance and lower interlaminar strength have been suspected to have spread out delamination, as perceived by profilometric oversize values (entry: 6200 µm; exit: 6280 µm). This confirms GFRP’s tendency to suffer thermal and force overload when cutting hole. SEM photos of B_4_G_6_B_4_ GFRP (Figure 8c) cut by HSS G show a combination of damage mechanisms—tearing out of fibers, peeling out of resin, and limited delamination around resin–ply interfaces. Force and temperature were moderately high (85.59 N and 104.4 °C, respectively), considering a hybrid laminate stacking of stiff basalt and soft glass plies. Profilometer results indicated entry and exit oversizes (6180 µm and 6250 µm, respectively), showing progressive damage by cutting tunnel while passing through laminate stack. SEM cross-section photograph of G_4_B_6_G_4_ after cutting by HSS-G exhibit widespread delamination by pushing out and pull-out of fibers, mostly on exit (Figure 8d). Thrust force was 69.8 N and max temperature while drilling was 111.2 °C. Hybrid lay-up reduced some severity of G_14_’s damage, but soft external glass layers and thermal overload led to widespread damage progression. Entry and exit diameters (6170 µm and 6240 µm) exhibited this progression.

**Figure 9:**
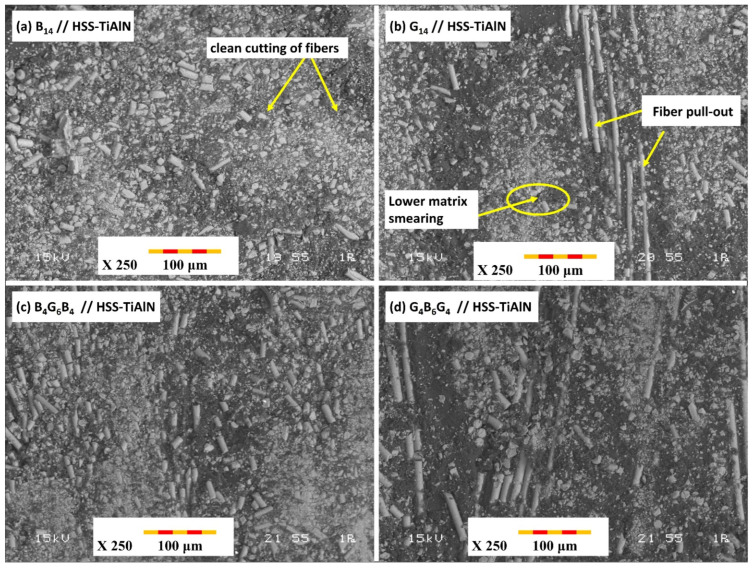
SEM images of the drilled hole cross-sections of the composites using the TiAlN-coated HSS drill bit (**a**) B_14_, (**b**) G_14_, (**c**) B_4_G_6_B_4_, (**d**) G_4_B_6_G_4_.

The SEM studies on cross sections of drilled holes using TiAlN-coated HSS drill bits show considerably better surface finish and lower damage characteristics for all three composition types (Figure 9). SEM micrograph of B14 composition drilled using TiAlN-coated bit shows clean cutting of fibers and minimum matrix deformation (Figure 9a). Both thrust force and temperature were considerably lower (26.59 N and 69.1 °C) while size accuracy was better (entrance: 5940 µm; exit: 6020 µm). Friction was reduced and heat accretion was avoided by TiAlN coating, hence good fiber–matrix integrity and a better cross section was produced. SEM characterization of G_14_ cross section using TiAlN-coated drill shows well-cut fibers and lower matrix smearing (Figure 9b). Moderate thrust force (27.71 N) and lower temperature (78.1 °C) ensured minimum thermal deterioration. Holes showed better geometrical shape (entrance: 5970 µm; exit: 6050 µm), hence confirming a positive role of TiAlN coating on reducing both its inherent/mechanical and thermal inputs, more specifically in composition rich in glass fibers. A cleaner and better-preserved matrix and fibers were visible on SEM using TiAlN drill (Figure 9c), with lower thrust force (29.77 N) and temperature (78.3 °C) signifying its insulating and drag-reducing actions. Holes’ diameters (entrance: 5960 µm; exit: 6030 µm) were maintained very near to their original values, hence confirming insignificant delamination and better size integrity, largely due to arresting effects by alternate plies of fibers. Cleaner hole faces, minimum fraying of fibers, and well-preserved matrix interface were visible on SEM (Figure 9d) using TiAlN-coated drill. Lower thrust force (26.16 N) and temperature (74.2 °C) reflect its very good tendency to restrain both its inherent/mechanical and thermal damages. Entrance and exit diameters (5960 µm and 6010 µm) were maintained almost nominal and hence confirmed its very good performance for its capability to retain structural integrity in hybrid laminates.

The SEM examination of the hole cross-sections drilled by B_14_, G_14_, B_4_G_6_B_4_, and G_4_B_6_G_4_ laminates indicated a clear correlation between induced damages and the nature of drill used, corresponding well to experimental measurements of thrust force, temperature, and hole size. In all materials, the HSS-G (uncoated ground HSS) drill showed by far the worst damages including fiber pull-out, matrix smearing, and interfacial cracking. This was most pronounced in G_14_ laminate containing lower interlaminar strength and high thermal sensitivity, which showed extensive delamination and hole oversizing (up to 4.7% on exit) diametrically. In sharp contrast, coated TiAlN exhibited best-in-class performance for all laminates by significantly reducing thrust force and thermal loading, particularly in B_14_ and hybrid laminates, allowing cleaner cuts of fibers and minimum matrix degradation and nearly nominal hole size. TiAlN’s performance can be attributed to its ability to reduce tool–work interface friction and consequently limit heat generation and suppress thermally induced failure mechanisms. Hybrid laminates (B_4_G_6_B_4_ and G_4_B_6_G_4_) showed moderate damage response as compared to their corresponding monolithic equivalents, hence indicating alternate basalt and glass plies can reduce crystallite growth and evenly distribute contact pressure while cutting. In both hybrid variants, however, SEM images showed reduced delamination and more acceptable hole cross section texture while using coated tools.

### 3.4. Factorial Analysis

Analysis of variance (ANOVA) is a powerful statistical method used in multifactorial experimental studies to quantitatively reveal the effect of each variable on the dependent variable and to determine whether these effects are statistically significant. Particularly in multivariate experimental setups evaluating the machinability of composite materials, ANOVA analysis allows us to determine which parameters are dominant in the process; this provides guidance for process optimization, tool selection, and material design. In this context, the contribution (%) value, which indicates the contribution of each factor to the total variance, is also of great importance. Contribution ratios clearly provide the researcher with a clear understanding of which parameter explains how much of the systematic variation. This allows us to prioritize which variables are most critical to control in practice. In this study, a detailed examination of contribution values in optimizing an output such as thrust force, which directly affects process performance, contributed to the accurate identification of the most significant factor affecting the mechanical load distribution of the drilling process.

The variance analysis applied to the values of thrust force in Table 2 tests the statistical significance of the type of drill bit and type of drill-bit coating on the applied thrust force during the drilling process. Analysis results show that both factors statistically affect the thrust force, although their statistical influence is quite different. The drill-bit factor contributed the maximum, representing 92.71% of the total variance. From the result, it is suggested that the type of drill bit used, that is, the type of its coat attributes, is the determinant factor under which the forces during the process of drilling are developed. This is in agreement with the previous results that drill bits with TiN and TiAlN, among others, result in low values of thrust force due to the reduction in the friction and thermal effects.

The second factor, “Material,” shared only 4.39% of the total variance, which indicates that composite structures (i.e., glass fiber, basalt fiber, or hybrid stacks) influence the force requirements in the process of drilling, but it is extremely negligible in contrast to the drill coat bit. High abrasiveness of the glass fiber is considered one of the overriding factors responsible for the observed difference. The error term, which contributed 2.90%, reveals limited variability and hence supports the repetitiveness and consistency of the experimental setup.

**Figure 10:**
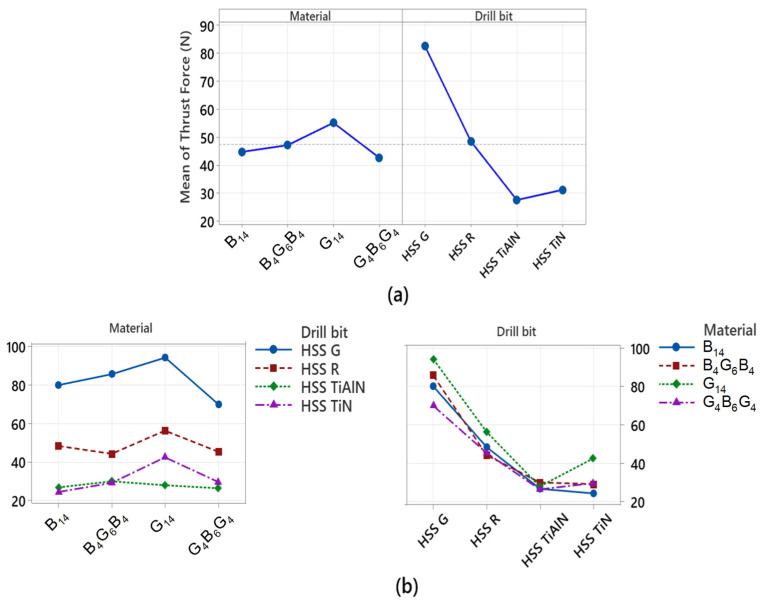
(**a**) Mean main effects and (**b**) interaction plot for thrust force.

Figure 10 shows how the factors affecting the thrust force measured during drilling vary individually (mean main effects) and together (interaction plots). The main effect plot in Figure 10a clearly demonstrates the dominant effect of the drill-bit factor on thrust force. Uncoated and ground HSS-G drills produce the highest average thrust forces, while TiN- and TiAlN-coated drills operate at significantly lower forces. This is graphically confirmed by the drill-bit factor, which has the highest contribution value (approximately 93%) in the ANOVA results. Similarly, the material variable also has an effect on thrust force, but the range of variation is narrower. G_14_ (all-glass fiber structure) stands out with its high thrust force requirement, while hybrid composites with arrays such as G_4_B_6_G_4_ exhibited lower average forces. This reflects the impact of the composite architecture on load distribution and cutting resistance. The interaction plot in Figure 10b presents the interrelationship between the drill bit and material factors. The non-parallel nature of the curves indicates a significant interaction between these two variables, indicating that the effect of drill bit type on thrust force varies depending on the composite material type. For example, the thrust force values obtained with the HSS-G bit in the G_14_ composite are significantly higher than those in other combinations, while this difference decreases significantly with TiAlN-coated bits. This demonstrates that coated drills provide a more significant performance improvement, particularly in abrasive and thermally sensitive materials such as glass fiber. In conclusion, Figure 10 is the pictorial representation of the results of the ANOVA, showing clearly that drill-bit coating technology is the most important factor in decreasing the force of drilling. Additionally, the necessity of the process parameters to be optimized collectively, considering the interaction between the composite structure and the type of the cutting tool, is underlined.

The variance analysis in Table 3 was carried out with the aim of finding out the factors that affect the surface temperature of the material (material temperature) during the process of drilling. Here, the type of composite material and the type of drill bit were used as the independent variables, while the average measure of the temperature observed around the drill was used as the dependent variable. From the results, the drill-bit factor accounted for the relatively large proportion of overall variance, which is 86.62%. This result clearly shows that the type of drill bit used; most importantly, its coating presence or otherwise, is the major determinant of the temperature of the material. This is in agreement with the existing findings that the coated bits (TiN and TiAlN) avoid the accumulation of thermal energy due to the minimization of the force of friction, hence regulating the rise in temperature at the surface of the examined materials. Here, the drill-bit factor has considerable influence, not only on the mechanical forces, but the thermal loads as well. Conversely, the material (composite structure) factor impacted very little, adding merely 1.73%, and the observed difference was statistically insignificant. This implies that the type of composite (glass fiber, basalt, or hybrid arrays) has a zero influence on the temperature of the material, or that the level of accumulated heat between the different structures is insignificant. This, in essence, implies that the thermal loads during the process of drilling are essentially the result of the force of the frictions at the interface between the tool and the materials, and the surface coating of the tool directly impacts the level of the frictions.

**Figure 11:**
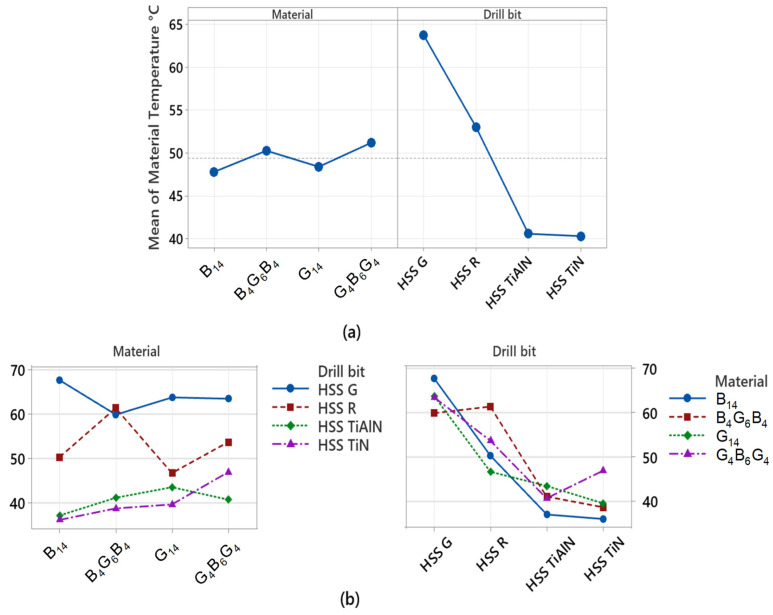
(**a**) Mean main effects and (**b**) interaction plot for material temperature.

Figure 11 shows how the temperatures occurring on the composite material surface during the drilling process change both independently (main effects) and interactively. An examination of the mean main effects plot in Figure 11a reveals that the drill-bit factor clearly has a dominant effect. Particularly, uncoated and ground HSS-G inserts significantly increased the material temperature, and TiN- and TiAlN-coated drills reduced the material temperature and limited the thermal effect. This shows that the coatings reduce the coefficient of friction and dampen the generation of heat in the contact interface between the material and the tool. This observation is consistent with the high contribution value of 86.62% for the drill-bit factor in the ANOVA results. On the other hand, the effects of the material variable are quite low, and no significant difference is observed between the plotted curves. This suggests that the material architecture (glass, basalt, or hybrid structure) does not directly contribute to the surface temperature; the temperature increase depends more on the thermal behavior of the tool. The interaction plot in Figure 11b reflects the interaction between the drill bit and material factors. The fact that the curves in the plot are quite close and mostly parallel indicates that there is no significant interaction between these two variables. In other words, the effect of drill bit type on material temperature is similar regardless of the type of composite used. This confirms that coated drill bits exhibit consistent thermal performance across all composite types.

The variance analysis that is reported in Table 4 assesses the factors affecting the temperature values developed in the drill body in the course of the drilling process. The drill temperature was taken as the dependent variable, and the type of composite and type of drill bit as independent variables during the analysis. The analysis is important in unveiling the parameters that govern the pile-up of heat at the interface between the tool and the workpiece material. From the results, it is observed that the drill-bit factor (type of drill bit) accounts for 85.40% of the total variance, which implies that drill-bit coatings influence the temperature rise of the drill bit directly. Specifically, it is possible to argue that the TiN and TiAlN based drills limit the heating of the tool due to their thermal conductivity and coefficient of friction being very low, and hence the thermal damage is minimized. The finding is further borne out experimentally with the very high drill temperature values registered by the uncoated HSS-G drill. Conversely, the material factor accounts for merely 8.57% of the total variance, and the result is statistically significant. This finding proves that the type of composite—specifically, the more abrasive and the ones that conduct the thermal loads poorly, such as the glass fiber—has a restricted but still considerable influence on the temperature of the drill bit. For instance, the higher drill temperature values registered in the structure with glass fiber density indicate that the material enhances the generation of the frictional heat. The error term accounts for merely 6.03% of the total variance, which may be taken as an encouraging result in the sense that the experimental system is very reliable and the data are highly consistent.

**Figure 12:**
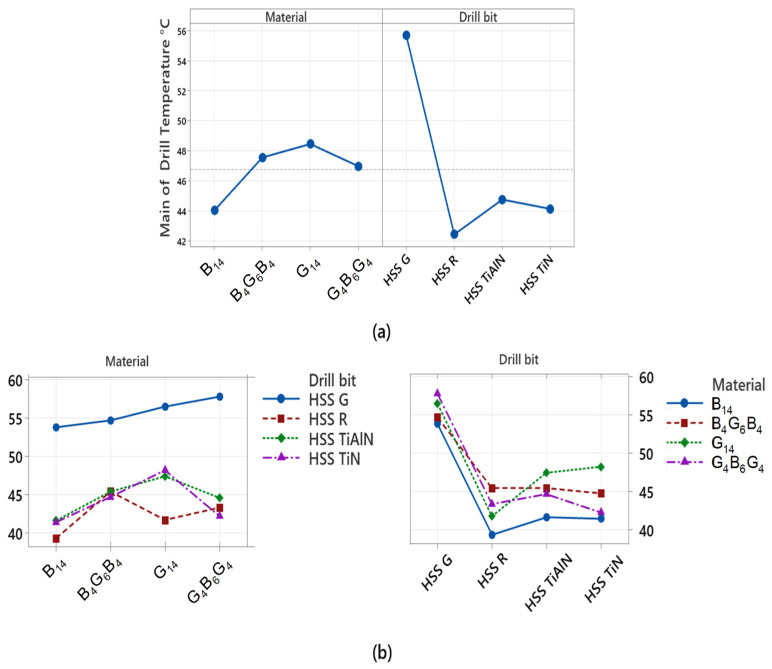
(**a**) Mean main effects and (**b**) interaction plot for drill temperature.

Figure 12 visually shows both the individual impacts (main effects) and the interaction plots (interaction plots) of the factors that determine the drill temperature during the process of drilling. This is a graphical representation of the results of the ANOVA and clearly shows which parameters result in the accumulation of heat in the tool body. Analyzing the mean main effects plot in Figure 12a, it is clearly seen that the most influential variable on the drill temperature is the drill-bit factor. The uncoated HSS G tip registered the maximum value of the mean temperatures, with TiN- and TiAlN-coated tips running at very much lower temperatures. This implies that physical vapor deposition coatings restrict the production of thermal energy at the tool–material interface by minimizing the level of friction. This finding is also in agreement with the statistical significance of the drill-bit factor in the ANOVA results, with 85.40% contribution. The curves representing the main effects of the material factor, on the other hand, show a flat trend, and the variations between them are quite insignificant. This indicates that the influence of the type of composite structure (G_14_, B_14_, B_4_G_6_B_4_, G_4_B_6_G_4_) on the drill temperature is restricted but not insignificant. The reason why structures with the higher glass fiber component, such as the type G_14_, result in higher heating of the tool may be attributed to the abrasive nature of the type of the material used. The interaction plot in Figure 12b is an assessment of the interaction between the drill bit and the material factors. Since the curves are near but not entirely parallel, it indicates that there is restricted interaction between the factors. This implies that the overall influence of the drill-bit coatings is the same regardless of the type of the composite, but that the influence becomes higher in certain types of the materials (e.g., structures with the glass fibers).

The ANOVA analysis presented in Table 5 statistically evaluates the factors influencing the maximum drill temperature. According to the findings, drill bit (drill bit type) is the most significant variable, explaining 95.39% of the total variance, clearly demonstrating the friction and heat accumulation reduction effect of TiN- and TiAlN-coated drills in particular. In contrast, the material (composite type) factor contributed only 1.50% and was found to be statistically insignificant. This suggests that different composite structures do not significantly affect the maximum temperature, with the primary effect stemming from tool properties. The total error variance was limited to 3.11%, supporting the reliability of the experimental data. Consequently, tool coating is the decisive factor in controlling the maximum temperature. Coated bits positively affect both tool life and hole quality by limiting thermal loads. The effect of material type is minor.

**Figure 13:**
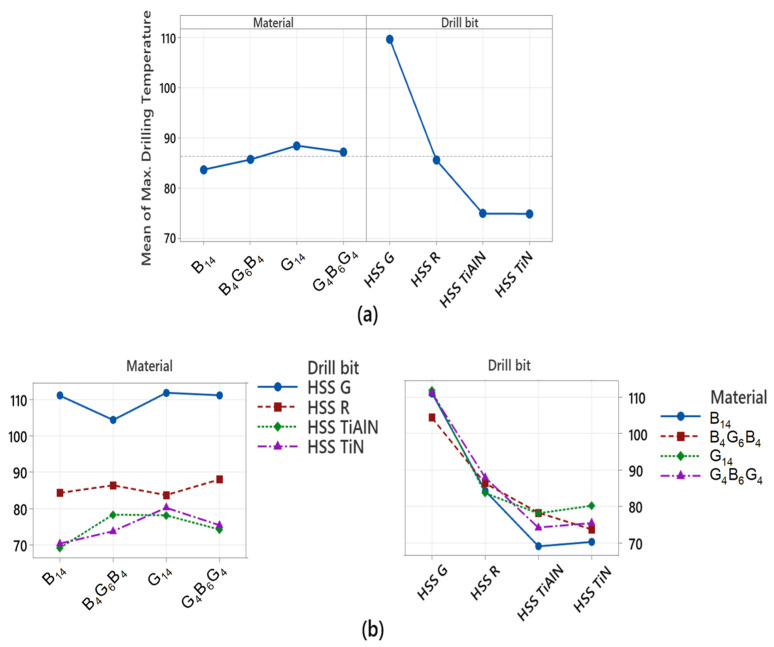
(**a**) Mean main effects and (**b**) interaction plot for maximum drilling temperature.

Figure 13 examines individually (main effects) and joint (interaction) effects of variables on maximum drill temperature during the cutting process. The figure provides visual proof of the statistical findings achieved through the use of ANOVA. The mean main effects plot in Figure 13a clearly indicates the dominant influence of the drill-bit factor on the maximum temperature. While the uncoated and ground HSS G drill provided the maximum temperature values, TiN- and TiAlN-coated drills reduced the maximum temperatures appreciably. This has the implication that the application of the coated drill reduces the coefficient of friction, which limits the accumulation of heat in the cutting zone and reduces the thermal confinement on the cutting tool. This finding is perfectly in agreement with the fact that the drill-bit factor contributed more than 95% during the ANOVA assessment. The curves showing the material factor are very close to each other, which indicates that the materials types do not play a significant role in the maximum temperature values. This has the implication that the maximum generator of the heat is the thermal and the friction characteristics of the cutting tool, and not the type of the material. The interaction plot from Figure 13b examines the interaction between the drill bit and the material factors. The parallel behavior of the curves indicates that there is no significant interaction between the variables, which has the implication that the effect of the drill bit type on the maximum temperature follows the same course in all the composite types.

**Figure 14:**
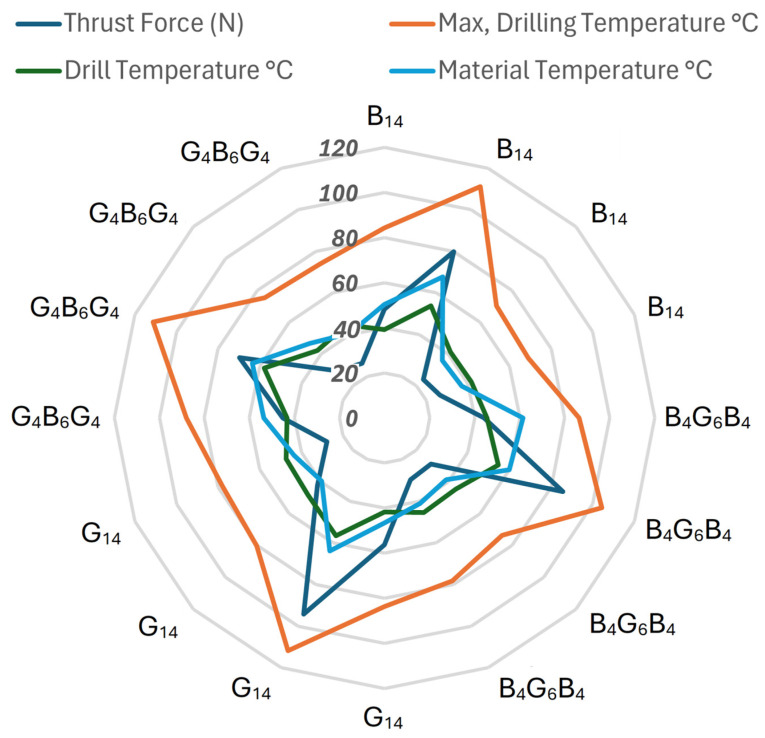
Radar chart comparing peak thrust force and drilling-induced temperatures (drill tip, drill body, workpiece) for G_14_, B_14_, B_4_G_6_B_4_ and G_4_B_6_G_4_ laminate configurations.

Figure 14 condenses the highest thrust force and thermal loads measured for each drill–material pair into a single radar plot, allowing the four laminates to be ranked at a glance. The all-glass fiber G_14_ laminate occupies the outermost envelope: it combines the highest thrust force (94 N) and the sharpest thermal peaks, reaching almost 120 °C at the tool tip and 57 °C in the drill body, while the surrounding work surface stabilizes at around 64 °C. These values are consistent with increased friction and difficulty in chip removal due to the abrasive nature of the glass fibers and the low interlayer strength, and are quantified in Table 1 and show that drill tip effects are dominant in the ANOVA, but laminate type is still an important secondary factor. The all-basalt B_14_ laminate follows with a thrust plateau of around 80 N and a peak temperature at the interface slightly exceeding 110 °C. The basalt layers’ higher modulus, yet greater stiffness, keeps the drill bit hotter, while the laminate dissipates heat more effectively, limiting the material-side temperature to 68 °C. Two hybrids exhibit a more compact signature: the basalt outer layers of B_4_G_6_B_4_ retain the thrust force at 86 N and rapidly dissipate heat, reducing the maximum temperature to 104 °C and the work-surface temperature to 60 °C; the G_4_B_6_G_4_ stack with a fiberglass outer layer exhibits the lowest mechanical demand of the four (70 N) but records a slightly higher material temperature (64 °C) due to the deep thermal insulation of the conformal glass layers. When the star-shaped polygons are considered together, it is clear that replacing the outer glass layers with basalt simultaneously stiffens the laminate and suppresses thermal dissipation; however, only coated drills can narrow the blue–green–orange footprint towards the chart center. This friction-reducing effect is the primary reason why drill bit selection trumps laminate architecture in the variance analysis. Figure 14 thus reinforces this study’s core message: while hybridization softens the negative edges of glass- or basalt-intensive designs, effective thermal management ultimately depends on a PVD-coated cutting edge with low cutting resistance.

**Figure 15:**
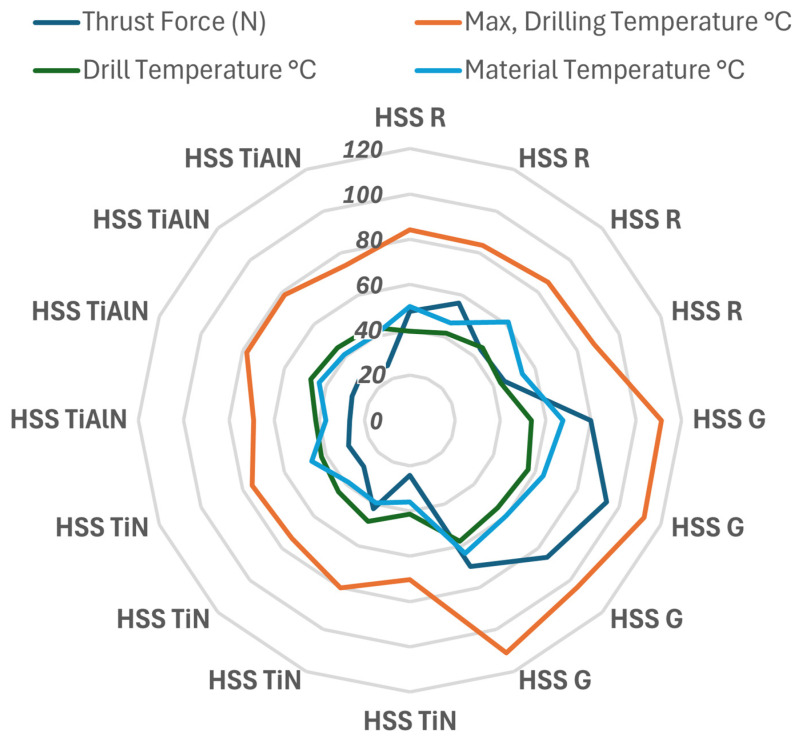
Radar chart contrasting average thrust force and peak drill-tip, drill-body and workpiece temperatures for uncoated HSS drills (HSS R, HSS G) versus PVD-coated variants (HSS TiN, HSS TiAlN).

Figure 15 contrasts the averaged thermal response and thrust force of four contrasted HSS drill designs: rolled (HSS-R), ground (HSS-G), and PVD-coated TiN and TiAlN inserts, in one single radar diagram, and extracts the essential trends in cutting performance. Uncoated inserts show the most unfavorable scenarios in both mechanical and thermal loading; the ground HSS-G insert, in particular, shows the most difficult cutting situation, spanning the greatest area of the graph with 70 N thrust force and 110–115 °C maximum temperature at the cutting-tool tip. Though the rolled, re-sharpened HSS-R insert moderates both loads to an extent (55 N, 90 °C), the temperatures of the body and the workpieces remain too high, with an enhanced danger of thermal softening and of hole-wall surface removal. PVD-coated inserts strongly constrain both the stick and the heat accumulation: the highly thermal-conductive TiN coat instantly evacuates the heat produced in the vicinity of the cutting chip-tool rake face, decreasing the cutting-tool temperature to 30 °C and the workpiece temperature to 40 °C and decreasing the thrust force to 40 N. Likewise, the hard TiAlN coat decreases the thrust force to below 40 N and holds the maximum temperatures in the 70–75 °C range. Its moderately lower thermal conductiveness partially retains the heat in the cutting edge, and since most of this energy is depleted thanks to the chip flow, the workpiece zone under the cutting zone does not overheat. Thus, Figure 15 clearly shows that the coated drills offer the tribological efficiency cutting down the thrust force between 25 and 40% and the critical peak temperatures 40 °C compared with their counterparts uncoated. Additionally, the very great TiAlN hardness, in particular, resists the dulling of the cutting edges, with the consequence of securing the cutting regime that is sustained.

### 3.5. Cutting Temperatures

**Figure 16:**
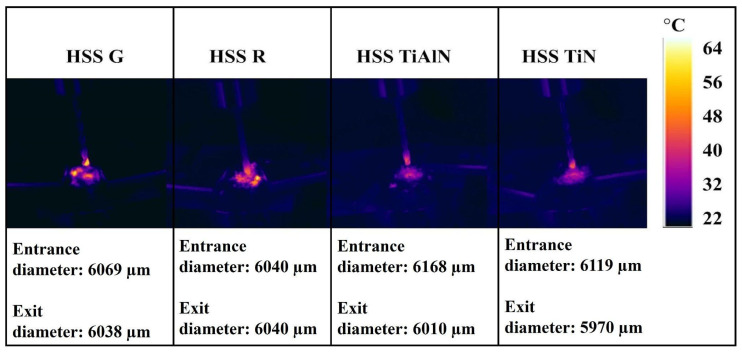
Temperature distribution images obtained for B_4_G_6_B_4_ according to different drill bits.

An infrared thermographic investigation of the B_4_G_6_B_4_ laminate during the process of drilling reveals that the drill type significantly affects the transfer and production of heat (Figure 16). The HSS G drill, among all the instruments examined, delivers a maximum temperature during the drilling process, which is 104.4 °C. High thermal output is experienced due to increased interfacial friction and the weak capability of the instrument to drive away the produced heat. The poor thermal management and uncoated nature of HSS G lead to the buildup of higher concentrations of warmth in areas near cutting edges. Furthermore, the higher drill temperature (54.7 °C) signifies the immense heating of the tool, which is an added cause of thermal transfer into the laminate. The HSS-R instrument, which is also uncoated, exhibited an inverse and moderately higher maximum temperature (86.4 °C) with thermal behavior relying heavily on inefficient thermal management and moderate level of material heating. Conversely, TiN- and TiAlN-coated instruments demonstrated notably smaller temperatures during the process of drilling. TiN drill exhibited the lowest overall values in the surface temperature of the material (38.7 °C) and the maximum temperature (73.7 °C) and, hence, has better thermal management. This is due to the fact that the application of the TiN coat decreases the level of frictions and facilitates the operation of the coat as the thermal barrier, hence restricted propagation of the generated heat into the laminate and the tool. TiAlN drill demonstrated exemplary thermal behavior with higher temperatures, albeit substantially yet higher, with respect to TiN and far below the uncoated instruments. Both the instruments exhibit compact and localized hot spots in the thermal capture, indicating more concentrated force transfer and limited thermal diffusivity. These thermal attributes agree with the results of thrust force, wherein the application of the coat revealed more settled cutting behavior. Low and regulated temperatures lower the chances of the matrix softening, the debonding of the fiber–matrix, and the peeling, which are all very important considerations for the maintenance of the hole quality and the structure’s integrity, especially in hybrid laminates such as the B_4_G_6_B_4_ laminate.

TiN coatings are known for their excellent hardness and low coefficient of friction, which reduces adhesion and frictional heating at the tool–workpiece interface, thereby lowering cutting temperature and mechanical loads. Their relatively good thermal conductivity facilitates localized heat dissipation, which helps reduce thermal softening during drilling. TiAlN coatings, on the other hand, exhibit superior oxidation resistance and thermal stability at elevated temperatures commonly encountered in dry drilling. This thermal stability allows TiAlN to maintain hardness and wear resistance under harsh conditions, especially during machining of abrasive, glass-rich composites. In glass-rich laminates, where abrasive glass fibers increase tool wear and frictional heating, TiAlN’s enhanced thermal resistance and hardness retention contribute to better tool durability and reduced delamination risk. While TiN coatings effectively reduce friction and heat generation, they may degrade more rapidly under these thermal and abrasive stresses.

**Figure 17:**
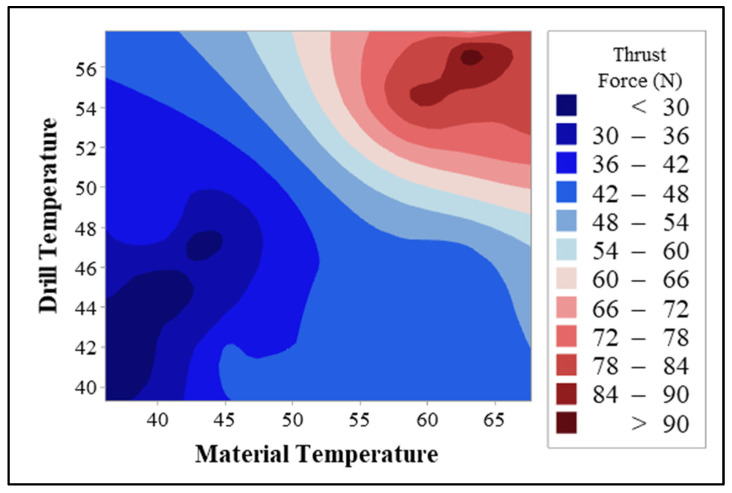
Contour plot of thrust force versus drill and material temperature.

Figure 17 is the contour plot created with the average drill and work material temperatures, which clearly shows the direct relationship between thermal states and cutting thrust force. At the lower temperature range, i.e., drill temperature is lesser than 46 °C and work material temperature is lesser than 48 °C, thrust force is maintained restricted, usually lesser than 42 N. This is the optimal cutting zone, wherein both thermal and mechanical loading remain highly manageable. As the temperatures increase, and in particular if the drill and work material temperatures exceed 54 °C and 60 °C, respectively, the thrust force is substantially greater, often greater than 90 N. This is an indicator of higher tool–material interaction, with higher thermal energy producing higher friction, probable matrix softening, and higher cutting resistance. The thermal energy apart from affecting the work material behavior is responsible for the deformation/wear of the cutting tool, which further increases the cutting load. This graph is also representative of the fact that drill temperature is more considerably affecting the thrust force as compared to work material temperature alone. The vertical gradients in the contour levels being greater compared to the horizontal ones signify that an increase, however minimal, in the drill temperature is indicating an appreciably larger increase in the thrust force. This further emphasizes the importance of effectively managing the generation of heat at the cutting interface so that mechanical efficiency is sustained and excessive loading is avoided during cutting processes.

## 4. Conclusions

The present work reveals that, with a fixed 1520 rpm/0.10 mm rev^−1^ drill regime, the cutting-edge tribology predominates the laminate structure in controlling force, thermal energy and damage, respectively, in the four laminates. Coating the same HSS drill with TiN or TiAlN damped effectively interfacial friction to divide applied thrust loads and freeze thermal peaks, and the lapped, Ti- and TiAl-coated-pro IPCC-G drill consistently generated the maximum mechanical (≥94 N) and thermal (≥110 °C) stresses. ANOVA attributed 92.7% and 86.6% of the variance in the thrust force and work-surface temperature, respectively, to the drill-bit factor and demoted the laminate type to secondary importance.

Quantitatively, the TiN and TiAlN coatings decreased average thrust from 79–94 N to 24–30 N, maximum interface temperature from 112 °C to 74 °C and entry/exit oversize from 2.5–4.7% to <0.6%. SEM confirmed these improvements, indicating clean fiber severance and intact epoxy walls, in sharp contrast with the fiber pull-out, matrix smearing and ply-edge delamination seen with uncoated tools. Hybrid lay-ups (B_4_G_6_B_4_, G_4_B_6_G_4_) balanced extremes by off-loading stiff basalt plies to flexible glass plies, but still relied on coated bits to achieve nearly nominal 6 mm diameters and minimum subsurface cracking.

Mechanistically, the PVD layers enhance surface hardness and form low-shear tribofilms that inhibit chip–tool adhesion, and hence, suppress the production of heat, matrix softening, and thrust-drawn ply separation. When thermal–mechanical inputs are reduced below the laminate interlaminar shear barrier, crack arrest in hybrids becomes effective, which clarifies their intermediate yet stable performance envelope.

For shop work, an industrially applied TiAlN- or TiN-coated 6 mm twist drill running on the tested feed–speed pair yields ±1% dimensional accuracy, hole-wall integrity with zero resin smear, and maximum workpiece temperatures far below the epoxy glass-transition range, specifications vital to long-term structural reliability. Future work should involve mapping wider feeds and spindle speed ranges of the performance of the coating, multi-hole wear cycles and life-cycle costs, and studies of assisted-cooling methods to extend the service life of the drill while still safeguarding composite integrity.

Table 6, distils the experimental trends into a concise, bullet-pointed set of practitioner-oriented guidelines—covering optimal TiN/TiAlN-coated tool selection, the validated 1520 rpm–0.10 mm rev^−1^ feed-speed window, and the associated ±1% dimensional-tolerance and <75 °C thermal thresholds—to support reliable, damage-free drilling of basalt–glass hybrid laminates in industrial applications.

Beyond mechanical and dimensional improvements, the use of TiN- and TiAlN-coated drills also contributes to more sustainable machining practices. Lower thrust forces imply reduced energy input per hole, while enhanced wear resistance leads to longer tool life and fewer replacements. These benefits collectively support waste reduction, resource efficiency, and lower environmental impact in composite manufacturing. From a cost-performance standpoint, TiN-coated drills stand out as a financially viable alternative to uncoated or grind-finished tools, offering a comparable price but significantly improved machining outcomes. Although TiAlN coatings are more expensive, their superior thermal and tribological properties may offset the cost in demanding or high-volume drilling environments. Therefore, selecting coating types can be tailored to operational priorities—cost-efficiency or extreme durability—without compromising dimensional integrity.

## Figures and Tables

**Table 1:** Experimental results.

Material	Drill Bit	Thrust Force (N)	Max. Drilling Temperature (°C)	Drill Temperature (°C)	Material Temperature (°C)
B_14_	HSS-R	48.21	84.3	39.3	50.3
G_14_	HSS-R	56.2	83.7	41.7	46.7
B_4_G_6_B_4_	HSS-R	44.12	86.4	45.4	61.4
G_4_B_6_G_4_	HSS-R	45.22	88	43.3	53.7
B_14_	HSS-G	79.9	111.1	53.8	67.7
G_14_	HSS-G	94.17	111.9	56.5	63.8
B_4_G_6_B_4_	HSS-G	85.59	104.4	54.7	59.9
G_4_B_6_G_4_	HSS-G	69.8	111.2	57.8	63.5
B_14_	HSS-TiN	24.19	70.3	41.4	36.1
G_14_	HSS-TiN	42.28	80.2	48.2	39.6
B_4_G_6_B_4_	HSS-TiN	28.94	73.7	44.7	38.7
G_4_B_6_G_4_	HSS-TiN	29.42	75.4	42.2	46.9
B_14_	HSS-TiAlN	26.59	69.1	41.6	37.1
G_14_	HSS-TiAlN	27.71	78.1	47.4	43.5
B_4_G_6_B_4_	HSS-TiAlN	29.77	78.3	45.4	41.1
G_4_B_6_G_4_	HSS-TiAlN	26.16	74.2	44.6	40.7

**Table 2:** ANOVA of thrust force.

Source	DF	Seq SS	Contribution	Adj SS	Adj MS	F-Value	*p*-Value
Material	3	355.8	4.39%	355.8	118.61	4.54	0.034
Drill bit	3	7518.2	92.71%	7518.2	2506.07	95.90	0.000
Error	9	235.2	2.90%	235.2	26.13		
Total	15	8109.2	100.00%				

**Table 3:** ANOVA of material temperature.

Source	DF	Seq SS	Contribution	Adj SS	Adj MS	F-Value	*p*-Value
Material	3	30.26	1.73%	30.26	10.09	0.45	0.726
Drill bit	3	1512.56	86.62%	1512.56	504.19	22.31	0.000
Error	9	203.43	11.65%	203.43	22.60		
Total	15	1746.24	100.00%				

**Table 4:** ANOVA of drill temperature.

Source	DF	Seq SS	Contribution	Adj SS	Adj MS	F-Value	*p*-Value
Material	3	44.03	8.57%	44.03	14.675	4.26	0.039
Drill bit	3	438.79	85.40%	438.79	146.265	42.46	0.000
Error	9	31.00	6.03%	31.00	3.444		
Total	15	513.82	100.00%				

**Table 5:** ANOVA of maximum drilling temperature.

Source	DF	Seq SS	Contribution	Adj SS	Adj MS	F-Value	*p*-Value
Material	3	50.63	1.50%	50.63	16.88	1.45	0.293
Drill bit	3	3220.24	95.39%	3220.24	1073.41	91.94	0.000
Error	9	105.07	3.11%	105.07	11.67		
Total	15	3375.94	100.00%				

**Table 6:** Practical drilling guidelines for basalt–glass hybrid laminates.

Parameter/Step	Recommended Setting	Verified Outcome	Industrial Note
**Drill bit**	6 mm TiAlN- or TiN-coated HSS twist drill	≤80 N thrust force; <75 °C hole-wall temperature	Coatings suppress frictional heating and flank wear, extending tool life to ~40 holes per edge
**Spindle speed**	1520 rpm	±1% entrance/exit diameter tolerance	Higher speeds (>1800 rpm) raise temperature >90 °C and promote resin smear
**Feed rate**	0.10 mm rev^−1^	Zero fiber pull-out; no inter-ply delamination	Feeds > 0.15 mm rev^−1^ double thrust and chip load, degrading surface integrity
**Coolant strategy**	Dry drilling; ambient air purge optional	Surface finish Ra < 1.6 µm; no moisture ingress	Liquid coolants are unnecessary and may swell the epoxy matrix
**In-process inspection**	Check diameter and burr height every 40 holes	Confirms stable dimensional accuracy and coating integrity	Replace or regrind tool when diameter drift exceeds ± 1%

## Data Availability

The datasets presented in this article are not readily available because the data are part of an ongoing study. Requests to access the datasets should be directed to the corresponding author.

## Abbreviations

The following abbreviations are used in this manuscript:

HSS-R	High-Speed Steel-Rolled
HSS-G	High-Speed Steel-Ground
HSS-TiN	High-Speed Steel coated with Titanium Nitride
TiAlN	Titanium Aluminum Nitride
ANOVA	Analysis of Variance
SEM	Scanning Electron Microscope
